# A Hybrid Ridge Regression–Convolutional Bidirectional Long Short-Term Memory Framework with Dual-Level Transfer Learning for State-of-Health Estimation of Lithium-Ion Batteries Under High Temperatures

**DOI:** 10.3390/ma19153332

**Published:** 2026-08-05

**Authors:** Chengwei Ge, Chunling Wu, Zhen Zhang, Kaile Cao, Li Wang, Mingwei Gao, Xiangming He

**Affiliations:** 1 School of Energy and Electrical Engineering, Chang’an University, Xi’an 710064, China; 2025138058@chd.edu.cn (C.G.); 2024901939@chd.edu.cn (K.C.); 2 Shaanxi Key Laboratory of New Transportation Energy and Automotive Energy Saving, School of Energy and Electrical Engineering, Chang’an University, Xi’an 710018, China; 3 Institute of Nuclear and New Energy Technology, Tsinghua University, Beijing 100084, China; wang-l@tsinghua.edu.cn; 4 Xi’an Stropower Technologies Co., Ltd., Xi’an 710076, China; mingwei.gao@stropower.com

**Keywords:** lithium-ion batteries, state-of-health estimation, high-temperature conditions, ridge regression, convolutional bidirectional long short-term memory, transfer learning

## Abstract

Accurate state-of-health estimation of lithium-ion batteries under high-temperature conditions (40–50 °C) remains challenging because of accelerated electrochemical degradation and strongly nonlinear aging patterns. This paper presents a hybrid Ridge regression–convolutional bidirectional long short-term memory framework with a dual-level transfer learning strategy. A Ridge regression baseline first captures the global degradation trend, after which a convolutional bidirectional long short-term memory network learns the nonlinear residuals. For cross-battery adaptation, Ridge coefficients are transferred through prior-regularized regression, and the pre-trained network is fine-tuned using limited target-domain data. The method is validated on cycling datasets from three institutions, namely Tsinghua University, the University of Oxford, and Tongji University, covering 15 batteries under temperatures up to 50 °C. Four health-related features are extracted and adaptively denoised using locally weighted scatterplot smoothing. In single-battery extrapolation, the proposed method achieves a root mean square error as low as 0.0009 on cell B6 at 50 °C, outperforming random forest, long short-term memory, bidirectional long short-term memory, and Ridge regression by 91.1%, 88.6%, 87.7%, and 82.0%, respectively. A cross-battery ablation experiment showed that the dual-level transfer learning strategy reduced the root mean square error from approximately 0.009 to 0.0028, whereas increasing network complexity alone yielded only marginal improvement. A further hierarchical ablation showed that jointly adapting the Ridge prior and the residual network achieved a mean RMSE of 0.004325, representing reductions of 9.39%, 4.14%, and 6.92% relative to the no-adaptation, Ridge-only adaptation, and residual-network-only adaptation configurations, respectively.

## 1. Introduction

The state of health (SOH) of lithium-ion batteries serves as a pivotal indicator of performance degradation. Beyond being essential for ensuring the safe and reliable operation of battery management systems, SOH estimation represents a primary research focus and a cutting-edge topic in battery state estimation [1]. However, limitations in the completeness and accuracy of online measurements, together with the nonlinear, time-varying, and uncertain nature of battery degradation, pose substantial challenges to accurate SOH estimation [2].

Current SOH estimation methods mainly fall into three categories: feature-based, model-driven, and data-driven approaches. Feature-based methods extract health-related features (HFs) from charge/discharge data that are closely linked to degradation mechanisms. Establishing a nonlinear mapping between HFs and SOH enables battery health estimation. To accurately capture the complex evolution of battery parameters and reduce environmental interference, multidimensional HFs must be extracted and integrated with data-processing models [3]. Consequently, single-feature approaches face considerable practical limitations.

Model-driven methods are built upon the physicochemical principles of lithium-ion batteries, allowing them to quantify aging patterns and estimate SOH with high accuracy. Electrochemical models (EMs) and equivalent circuit models (ECMs) are the most widely used in this category. EMs can precisely describe multi-step internal reactions, enabling mechanism-based estimation [3]. For example, the Doyle–Newman pseudo-two-dimensional model offers strong interpretability and high estimation accuracy. However, standard EMs involve numerous nonlinear equations, leading to high computational cost [4]. In contrast, ECMs are simpler in structure—typically composed of resistors, capacitors, and voltage sources—and can also support SOH estimation [5]. For instance, Fang et al. [6] employed a recursive least-squares method with a forgetting factor to identify ECM ohmic resistance online, thereby achieving SOH estimation. As the above analysis shows, the accuracy of model-driven SOH estimation depends heavily on model complexity and parameter identification, posing challenges for real-world implementation.

Advances in information technology and artificial intelligence have promoted data-driven methods to the mainstream for lithium-ion battery SOH prediction. By leveraging machine learning and deep learning, these methods extract aging information from sensor-collected parameters, construct mappings between HFs and SOH, and enable fast, accurate SOH prediction [7]. The effectiveness of data-driven approaches thus hinges on the efficient extraction of HFs and the adaptive selection of learning models [8]. Commonly used techniques include neural networks, support vector machines, and Gaussian process regression [9]. For example, Zhang et al. [10] adopted a long short-term memory (LSTM) network for SOH estimation, achieving high accuracy under varying conditions. Guan et al. [11] incorporated an attention mechanism and proposed a probabilistic sparse self-attention LSTM model, which reduced prediction errors compared to a plain LSTM. Li et al. [12] developed a multidimensional HF-based particle swarm optimization (PSO) algorithm to enhance LSTM performance, showing improved generalization over a conventional PSO-LSTM. To further boost accuracy, researchers have also explored multi-model fusion. Hu et al. [13] combined LSTM’s strength in local feature extraction with the Informer’s ability to capture global dependencies via a serial LSTM-Informer network. Wu et al. [14] introduced a cascade model integrating a convolutional neural network, LSTM, and a Kolmogorov–Arnold network, which outperformed single-network architectures.

Existing studies have primarily focused on SOH estimation under ambient operating conditions, while the performance of lithium-ion batteries under high-temperature conditions remains insufficiently investigated. At 40–50 °C, accelerated parasitic reactions at the electrode–electrolyte interfaces promote the growth and reconstruction of the solid electrolyte interphase and cathode electrolyte interphase, accompanied by intensified electrolyte decomposition. These reactions consume cyclable lithium and electrolyte components, thereby contributing to loss of lithium inventory and available-capacity fade. Meanwhile, interphase thickening, electrolyte depletion, and deterioration of electrode contact impede ionic and electronic transport, resulting in increased internal resistance and polarization. Depending on the electrode chemistry and cycling protocol, structural degradation and loss of active material may also contribute to long-term capacity decay. The coupling of these degradation processes leads to increasingly nonlinear and cell-dependent aging trajectories under high-temperature conditions [15]. In addition to these coupled degradation mechanisms, battery monitoring data collected under high-temperature conditions are often affected by sensor noise and external disturbances, which may obscure degradation-related information and deteriorate SOH estimation accuracy. Although numerous data-driven approaches have been developed for SOH estimation, their ability to simultaneously accommodate noisy measurements, nonlinear degradation dynamics, and cell-to-cell variations under high temperature conditions remains limited. Therefore, the objective of this study is to develop an accurate and transferable SOH estimation framework capable of addressing noise interference, nonlinear degradation dynamics, and cell-to-cell variations under high temperature aging conditions.

To achieve this objective, this study proposes a high-temperature SOH estimation framework based on adaptive denoising and hybrid deep learning. The main contributions are summarized as follows:

(1) A locally weighted scatterplot smoothing (LOESS)-based denoising strategy is introduced to suppress measurement noise in the extracted HFs while preserving their long-term degradation trends. A fixed and reproducible smoothing configuration is applied consistently to all batteries, thereby avoiding battery-specific or test-set-dependent parameter adjustment.

(2) A hybrid Ridge regression–convolutional bidirectional long short-term memory (Ridge-Conv-Bi-LSTM) model integrated with a dual-level transfer learning strategy is proposed for SOH estimation. By jointly transferring global degradation knowledge and nonlinear residual features, the proposed strategy reduces the root mean square error (RMSE) from approximately 0.009 for the non-transfer variants to 0.0028 in the cross-battery ablation task, demonstrating the effectiveness of dual-level knowledge transfer under limited target-domain calibration data.

(3) Comprehensive validation is conducted under high-temperature conditions (40 °C, 45 °C, and 50 °C). Experimental validation under single-battery extrapolation conditions shows that the proposed method achieves an RMSE of 0.0009 on B6 at 50 °C and 0.0023 on D6 at 45 °C, representing reductions of up to 91.1% and 81.3%, respectively, compared with the benchmark models. These results demonstrate the accuracy of the proposed framework under the high-temperature aging conditions.

## 2. Data Sources and Feature Extraction

### 2.1. Experimental Datasets and Preprocessing

The battery-aging data analyzed in this study were obtained from three sources. The Tsinghua dataset was generated by the authors’ research team at the Institute of Nuclear and New Energy Technology, Tsinghua University, through cycle-aging experiments on 6 Ah lithium iron phosphate pouch cells. These data have not been deposited in a public repository. The Oxford data were selected from the publicly available Oxford Battery Degradation Dataset 1 [16], which contains long-term cycling records of 740 mAh lithium-ion pouch cells. The Tongji data were obtained from the battery-aging experiments reported by Zhu et al. [17]. Detailed cell specifications, cycling protocols, operating temperatures, cell identifiers, and data-access information are summarized in Table S1 of the Supporting Information (SI).

Due to sensor measurement errors and external environmental interference, some cycles in the battery’s cyclic aging test produced extreme abnormal data, thereby reducing the reliability of HF extraction. Therefore, this paper performs preprocessing to remove abnormal cycle data before HF extraction.

### 2.2. Extraction of Health-Related Features

Within data-driven state-of-health estimation, health-related features should not only exhibit sensitivity to battery degradation but also retain interpretable relationships with the underlying aging behavior [18]. In this study, the selected health-related features are treated as measurable macroscopic indicators of battery aging rather than unique signatures of individual electrochemical reactions. They mainly characterize two coupled consequences of aging: available-capacity loss and resistance/polarization growth.

At high temperatures, accelerated interfacial side reactions, electrolyte degradation, and possible loss of active material jointly alter the voltage response, charge acceptance, discharge behavior, and internal resistance of the cell. Accordingly, four complementary health-related features are extracted from the charge–discharge data: HF1, the charge time required for the terminal voltage to rise from 3.30 to 3.45 V under constant-current charging; HF2, the internal resistance estimated from the voltage response to a current step; HF3, the characteristic voltage extracted during constant-current discharge; and HF4, the partial charge capacity within the 3.30–3.50 V interval. These time-, resistance-, voltage-, and partial-capacity-based features provide descriptions of high-temperature degradation. Their selection was based on three considerations: sensitivity to battery aging, consistent availability across the three datasets, and extractability from partial operating segments without electrochemical measurement equipment. Their definitions, extraction procedures, and interpretations are described below.

(1) HF1: Charge Time within a Fixed Voltage Window

The charge time within a fixed voltage window is defined as the time required for the terminal voltage to rise from an initial threshold to a target threshold under constant-current charging. Because this feature can be extracted from a partial charging segment without requiring a complete charge or discharge cycle, the time required for the terminal voltage to increase from 3.30 to 3.45 V is selected as HF1, as illustrated in Figure S1 [18].

From a physical perspective, high-temperature aging accelerates electrode–electrolyte interfacial side reactions and electrolyte degradation. The associated loss of lithium inventory and possible loss of active material reduce the accessible capacity within the selected voltage window, while resistance and polarization growth increase the terminal voltage at a given charge throughput under the same charging current. Consequently, the upper voltage threshold is generally reached earlier as aging progresses, resulting in a decrease in HF1. Therefore, HF1 reflects the coupled macroscopic effects of available-capacity loss and charging-polarization growth rather than serving as a unique signature of any single electrochemical degradation mechanism.

(2) HF2: Pulse-Derived Internal Resistance

HF2 is calculated from the terminal-voltage response when the battery transitions from the preceding rest period to constant-current discharge. Specifically, it is defined as the ratio of the voltage change to the corresponding current change, as expressed in Equations (1)–(3).

(1) $$∆V=\left|V_{1}-V_{0}\right|$$

(2) $$∆I=\left|I_{1}-I_{0}\right|$$

(3) $$R=\frac{∆V}{∆I}$$

In Equations (1)–(3), $V_{0}$ and $I_{0}$ denote the terminal voltage and current at the end of the rest period immediately before discharge, whereas $V_{1}$ and $I_{1}$ denote the corresponding values at the first sampling instant after constant-current discharge begins. $∆V=\left|V_{1}-V_{0}\right|$ and $∆I=\left|I_{1}-I_{0}\right|$ are the magnitudes of the voltage and current changes, respectively, and R=∆V/∆I is the pulse-derived internal resistance, expressed in ohms.

HF2 represents the apparent internal resistance derived from the short-time terminal-voltage response to the applied current step. During high-temperature aging, continued growth and reconstruction of electrode–electrolyte interphase films, electrolyte degradation, deterioration of ionic transport, and possible loss of electrical contact may increase the resistance of the cell. The resulting resistance growth produces a larger short-time voltage change under the same current step. Consequently, HF2 generally increases as the available capacity and SOH decrease, enabling it to characterize resistance growth and the associated reduction in power capability. Because the measured value depends on the voltage-sampling interval, HF2 should be regarded as a lumped pulse-derived electrical indicator rather than a pure electrochemical parameter, and it cannot independently separate the contributions of ohmic resistance and rapid polarization processes.

(3) HF3: Characteristic Voltage during Constant-Current Discharge

HF3 is defined as the terminal voltage measured at a predefined point during constant-current discharge. As battery cycle count increases, voltage drops more rapidly at the same discharge current, causing the termination voltage to be reached earlier. As shown in Figure S2, the measured constant-current discharge termination voltage exhibits a significant positive correlation with battery aging degree, thus serving as battery health-related feature HF3.

(4) HF4: Partial Charge Capacity within a Fixed Voltage Window

HF4 is defined as the charge delivered during constant-current charging while the terminal voltage rises from 3.30 to 3.50 V [18]. In this study, the charge-capacity increment within the 3.30–3.50 V interval is selected as HF4.

From a physical perspective, high-temperature aging can induce loss of lithium inventory and possible loss of active material, thereby reducing the accessible charge within the selected voltage window. Meanwhile, resistance and polarization growth shift the charging voltage–capacity relationship, causing the upper voltage threshold to be reached after a smaller amount of charge has been supplied under the same charging current. Consequently, HF4 generally decreases as the available capacity and SOH decline. Therefore, HF4 serves as a partial-capacity indicator of the coupled effects of capacity loss and polarization-induced voltage-curve displacement rather than a unique signature of any single electrochemical degradation mechanism.

The selected features were preferred over conventional incremental capacity analysis (ICA), differential voltage analysis (DVA), and electrochemical impedance spectroscopy (EIS)-based indicators primarily because this study focuses on online-oriented SOH estimation and cross-dataset validation. ICA and DVA can provide more mechanism-oriented diagnostic information through characteristic changes in the *dQ*/*dV* and *dV*/*dQ* curves. However, their derivative calculations amplify measurement fluctuations, and reliable feature extraction generally requires sufficiently smooth and comparable constant-current voltage–capacity curves covering the relevant characteristic regions. The locations and amplitudes of the extracted peaks may also be affected by current rate, temperature, resistance growth, and polarization [19,20].

EIS-based indicators provide frequency-dependent information related to ohmic, interfacial, and transport processes and can therefore offer stronger electrochemical diagnostic capability. Nevertheless, impedance measurements require dedicated excitation and acquisition equipment, controlled measurement conditions, and consistent state-of-charge and temperature settings; the measured impedance also varies with operating condition and measurement protocol [20,21]. In contrast, HF1–HF4 can be extracted directly from partial charge–discharge segments and current-step responses using routinely measured voltage, current, and time signals, without numerical differentiation or additional impedance-measurement hardware [18]. This improves their computational simplicity, cross-dataset consistency, and suitability for online and cross-battery SOH estimation. The trade-off is that the selected features have lower mechanism specificity: they characterize the coupled macroscopic consequences of capacity loss and resistance/polarization growth but cannot uniquely distinguish loss of lithium inventory, loss of active material, interphase-film growth, or electrolyte degradation. In particular, HF2 is a lumped pulse-derived resistance indicator and does not provide the frequency-resolved process separation available from EIS. Therefore, the selected features are intended as practical and complementary SOH indicators rather than substitutes for dedicated electrochemical diagnostic techniques.

### 2.3. LOESS-Based Denoising of Hfs

**Figure 1:**
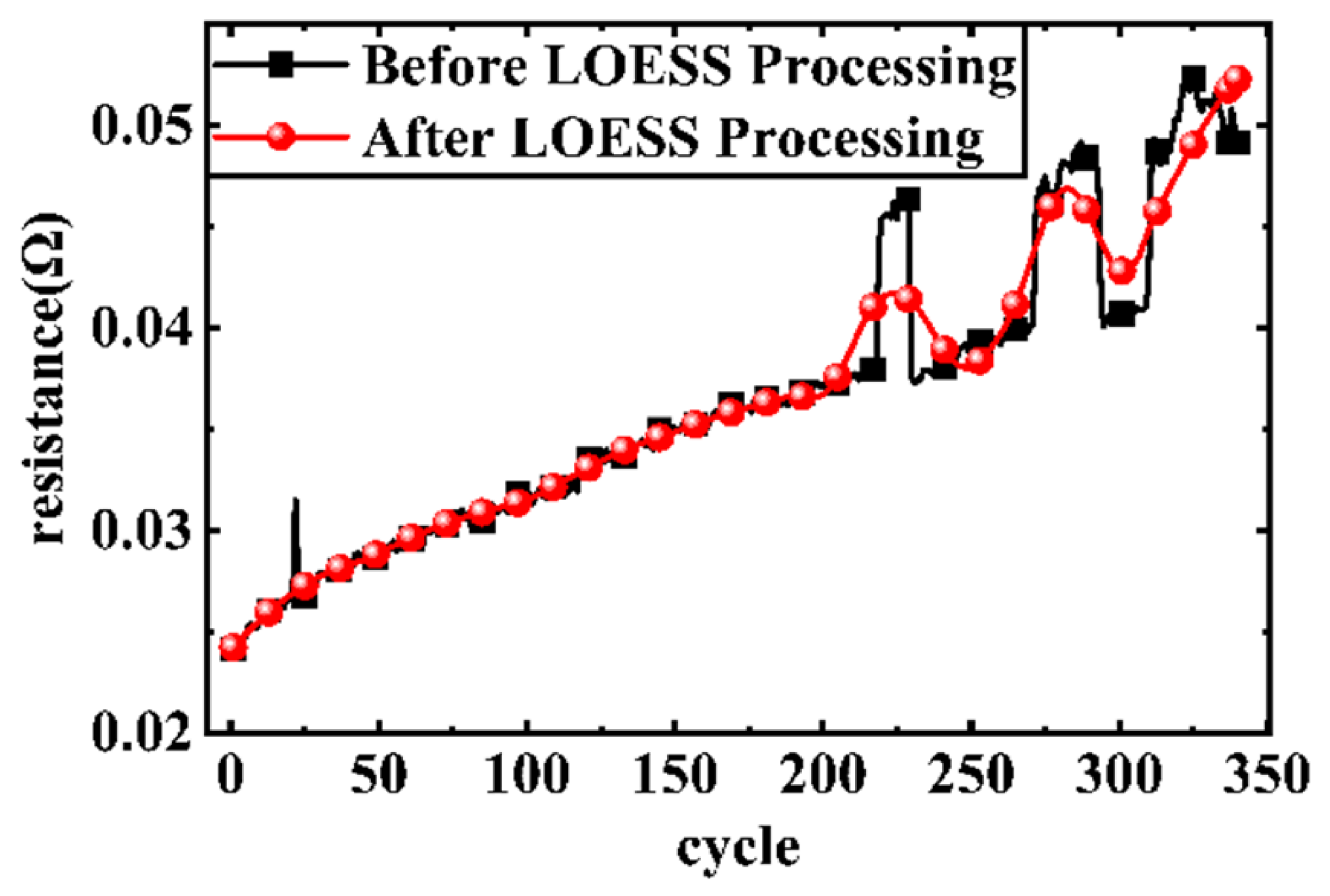
Comparison of pulse-derived internal-resistance values before and after LOESS processing.

The four HF sequences extracted in this study exhibit significant noise interference. Directly using them as model inputs would result in substantial estimation errors. To enhance the reliability and effectiveness of the feature data, LOESS is employed to perform denoising of the HF sequences, thereby filtering out noise interference and optimizing data quality. LOESS is a data denoising and smoothing algorithm. By setting the filter window width, it can rapidly filter original signals containing noise and spike interference while preserving the overall evolutionary patterns of the original data [22]. Following the 3σ outlier-removal step, the normalized LOESS span was fixed at 0.2 for all four HFs and all battery datasets. This setting means that approximately 20% of the valid observations are included in each local fit, while the actual neighborhood width varies with the distance to the corresponding nearest samples. A moderate span of 0.2 was selected to balance noise suppression and trend preservation: a smaller span is more sensitive to isolated measurement fluctuations, whereas a larger span may excessively smooth local changes in the degradation trajectory. The setting was kept unchanged across batteries rather than being optimized separately for individual datasets or prediction results. The mathematical formulation and implementation details of the LOESS procedure are provided in the Supporting Information. The outcomes of implementing LOESS to HF2 are shown in Figure 1. The figure demonstrates that the feature curve processed by LOESS is smoother than the original curve, effectively reducing the impact of noise interference on the HFs. The detailed denoising process is described in Supplementary Information.

### 2.4. Correlation Analysis via Pearson Coefficient

To validate the strong correlation between the extracted HFs and battery capacity, the relationship between the two was quantified using Pearson’s correlation coefficient. The battery HF dataset is denoted as *X*, and the capacity dataset as *Y*. The correlations between the selected HFs and battery capacity are quantified using the Pearson correlation coefficient, as expressed in Equation (4) [23].

(4) $$\rho_{XY}=\frac{Cov(X,Y)}{\sqrt{D(X)}\sqrt{D(Y)}}=\frac{E(XY)-E(X)E(Y)}{\sqrt{D(X)}\sqrt{D(Y)}}$$

In the formula, *D* denotes variance and *E* denotes mean. The complete correlation coefficients for all cells are provided in Table S2 of the SI.

The results show that HF1 and HF4 exhibit consistently strong positive correlations with capacity, with correlation coefficients ranging from 0.9699 to 0.9988 and from 0.9742 to 0.9985, respectively. This indicates that the charge time and partial charge capacity within the selected voltage windows generally decrease as the available capacity declines. In contrast, HF2 and HF3 are negatively correlated with capacity, with absolute correlation coefficients ranging from 0.7976 to 0.9986 and from 0.7699 to 0.9972, respectively. Their wider variation across cells suggests that the resistance- and discharge-voltage-related responses are more sensitive to differences in cell chemistry, cycling protocol, and degradation trajectory. Overall, the four features provide complementary information on capacity loss and resistance/polarization growth and are therefore retained as inputs to the subsequent SOH estimation model.

## 3. The Proposed Ridge-Conv-Bi-LSTM Framework

### 3.1. Ridge Regression Baseline

Ridge regression is a biased estimation method that introduces an L2 regularization term into least-squares estimation and can be regarded as a regularized extension of linear regression [24]. This method addresses situations where there is high correlation among independent variables (i.e., multicollinearity). By adding an L2 norm penalty term for the coefficient vector to the objective function, it constrains model complexity, thereby improving the stability and generalization ability of the coefficient estimates [25].

The general form of regression analysis is as shown in Equation (5).

(5) $$y=\sum_{j=1}^{p}\beta_{j}x_{j}+\beta_{0}$$

In the Equation, $x_{j}$ is the predictor for the jth feature, *j* = 1, 2, 3, …, p; *y* is the observed variable; $\beta_{j}$ is the parameter to be determined; $\beta_{0}$ is the error.

The objective of using the least squares method to solve the above regression problem is to obtain the regression coefficients, as shown in Equation (6):

(6) $$\beta =\arg\underset{\beta }{\min}\sum_{i=1}^{p}{\left(y_{i}-\beta_{0}-\sum_{j=1}^{p}\beta_{j}x_{ij}\right)}^{2}$$

In the Equation, $y_{i}$ is the observed variable for sample *i*, $x_{ij}$ is the predicted value for the jth feature of sample *i*; *N* is the sample size.

Based on Equation (6), a penalty term is added to the minimization objective to obtain the ridge regression coefficients, as shown in Equation (7).

(7) $$\beta^{bridge}=\arg\underset{\beta }{\min}\sum_{i=1}^{N}\left[{\left(y_{i}-\beta_{0}-\sum_{j=1}^{p}\beta_{j}x_{ij}\right)}^{2}+\lambda \sum_{j=1}^{p}\beta_{j}^{2}\right]$$

In the Equation, λ is the parameter to be estimated. Ridge regression is a least-squares regression with an L2 regularization term.

This paper employs Ridge regression to construct a linear degradation benchmark for SOH. Using normalized HF as input, it fits the overall trend of capacity degradation through polynomial feature expansion and L2 regularization. This benchmark provides an anchor for subsequent residual learning, enabling the Conv-Bi-LSTM network to fit only the nonlinear deviation between the true SOH and the Ridge-based prediction, thereby reducing the fitting burden on the deep network. Additionally, during the multi-branch fusion stage, the linear prediction branch derived from Ridge provides low-frequency degradation components to the overall output, complementing the deep residual branch and mitigating the risk of overfitting under sparse data conditions.

### 3.2. Conv-Bi-LSTM Residual Network

Convolutional neural networks achieve multi-level feature extraction through convolution operations, local connections, and weight sharing mechanisms, enabling them to effectively capture local structural patterns and contextual information in data [26,27]. In recent years, the application of one-dimensional convolution to time-series feature modeling has become a hot topic in the field of sequence analysis [28]. This paper introduces a one-dimensional convolutional layer at the front end of the Bi-LSTM network to extract local temporal patterns from multidimensional degraded feature sequences. This layer slides along the time dimension to capture local correlations among augmented inputs, including the normalized HFs, their derived differences and deviations, cycle indices, and Ridge basis functions. It provides more discriminative sequence representations for the subsequent Bi-LSTM, thereby enhancing the network’s ability to model the nonlinear degradation of battery capacity.

As a significant branch of recurrent neural networks, LSTM was specifically designed to mitigate the vanishing gradient and exploding gradient problems encountered by traditional RNNs in processing sequential data. Its innovation lies in introducing a gating mechanism and memory cells with selective recall capabilities: the gating mechanism precisely regulates the transmission and filtering of information flows, while memory cells adaptively retain crucial temporal information and discard redundant noise. The network’s synergistic operation facilitates the efficient extraction of long-term dependencies within sequential data, thereby circumventing the technical limitations of conventional RNN in handling long-term dependency tasks. This provides an effective solution for feature learning in complex time series [29]. For detailed information regarding the LSTM computation process, please refer to Supplementary Information.

**Figure 2:**
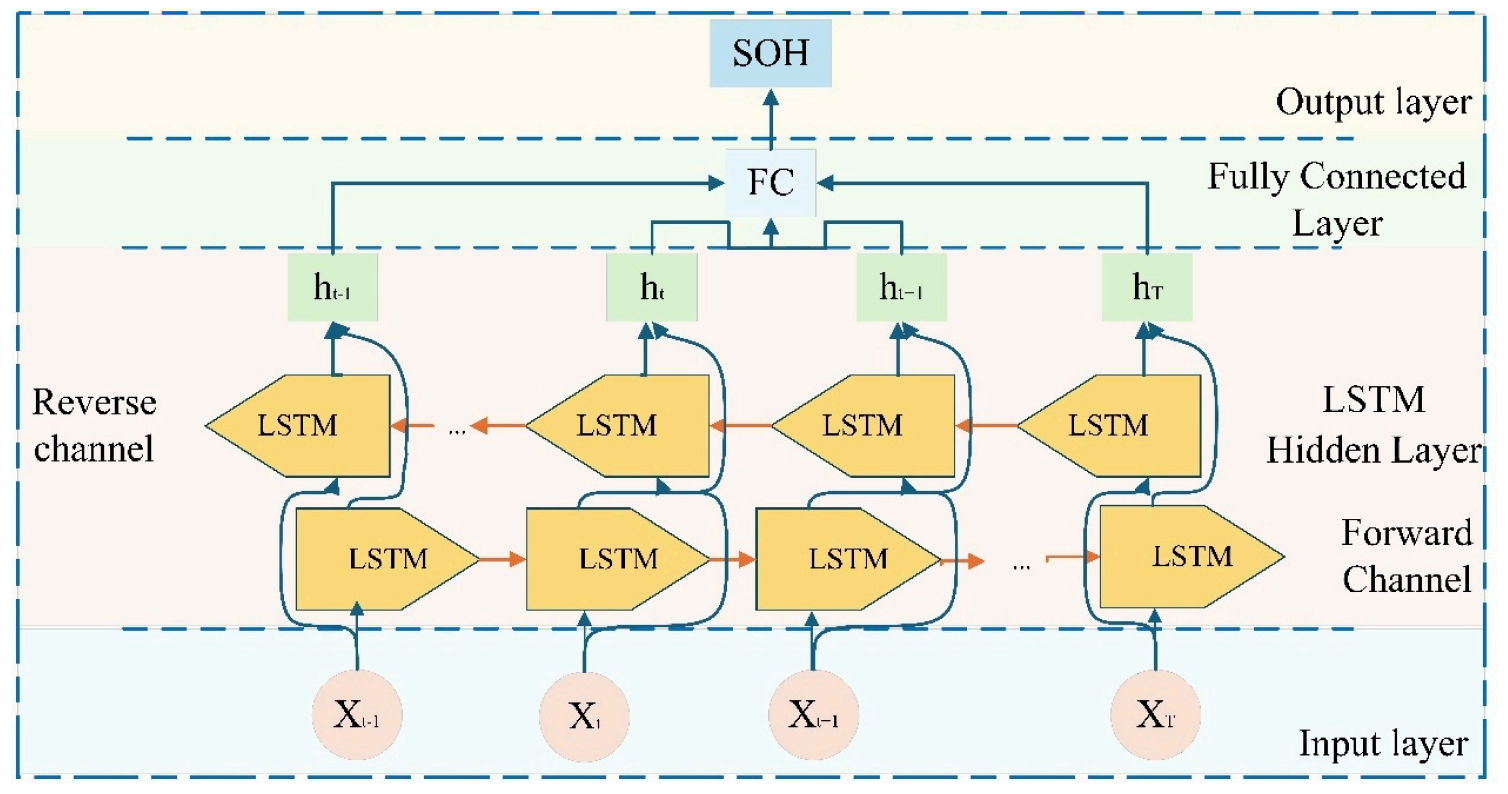
Schematic structure diagram of Bi-LSTM.

Although unidirectional LSTM effectively mitigates gradient explosion issues, their inherent structural limitations restrict them to capturing only forward dependencies in sequence data. Consequently, certain features related to battery aging are filtered out by the gating mechanism, ultimately reducing the estimation accuracy of battery SOH [30]. To address the inherent limitations of LSTM, this study introduces the Bi-LSTM model. This model consists of two structurally independent LSTM layers: As demonstrated in Figure 2, the initial sequence is processed in forward chronological order, whilst the subsequent sequence is processed in reverse chronological order [31]. This bidirectional architecture captures long-range dependencies in both forward and reverse directions, substantially enhancing the model’s ability to extract temporal features from data.

### 3.3. Dual-Level Transfer Learning Strategy

When the training battery and the test battery are different units, individual variations in capacity degradation trajectories lead to distribution shifts when the source-domain model is applied directly; moreover, due to the limited amount of data from the early stages of the target battery, it is difficult to model it independently. To address this, this paper performs parameter transfer on both the linear degradation benchmark and the deep residual network: the Ridge coefficient uses the source-domain estimate as a prior to guide the target-domain fitting; the Conv-Bi-LSTM network is initialized with pre-trained weights and fine-tuned on the calibration segment. The two transfer mechanisms are described in detail below. The proposed strategy differs from conventional single-level transfer learning in the object being transferred. Conventional approaches generally fine-tune a single end-to-end nonlinear mapping from battery features to SOH. Here, the SOH mapping is first decomposed into a global trend component and a nonlinear residual component. The source-domain Ridge coefficients provide an explicit prior for adapting the global degradation trajectory, whereas the pre-trained Conv–Bi-LSTM weights transfer the representation of local nonlinear residual dynamics. Therefore, the two stages address different sources of cross-battery error rather than duplicating the same adaptation operation.

The Ridge coefficients are migrated using prior-regularized regression for cross-battery adaptation of the linear baseline. Let the source domain Ridge coefficient be $\beta_{source}$, and let the design matrix and SOH observation values for the target battery calibration segment be $\Phi_{cal}$ and $y_{cal}$, respectively. The target domain coefficient $\beta_{t\arg et}$ is obtained via Equation (8).

(8) $$\beta_{t\arg et}=\arg\underset{\beta }{\min}\left\{{\left\|y_{cal}-\Phi_{cal}\beta \right\|}^{2}+\lambda_{prior}{\left\|\beta -\beta_{source}\right\|}_{I^{*}}^{2}\right\}$$

In the Equation, $\lambda_{prior}$ represents the prior regularization strength, and $I^{*}$ is the identity matrix with the elements corresponding to the intercept term set to zero. This penalty term, guided by $\beta_{source}$, constrains the direction and magnitude of coefficient estimates with a small number of calibration samples, suppresses overfitting, and enables the transfer of degradation trends across individuals.

In contrast to domain-adaptation methods, the fine-tuning strategy used here does not explicitly align distributions. Fine-tuning strategies do not directly compare the distribution differences between the source and target domains. They are based on the premise that the tasks in both domains are identical, requiring only minor adjustments to the model’s representation of the target data’s fine-grained features. This strategy initializes the target-domain model using source-domain parameters and subsequently updates the network using a limited number of labeled target-domain samples [32]. The transfer of the Conv-Bi-LSTM network in this paper adopts this strategy: after pre-training in the source domain is complete, the obtained network weights are used as the initial values for the target network, and training continues on the target battery calibration sequence. The network structure remains unchanged during training; the initial learning rate is reduced to 2 × 10^−4^ and the L2 regularization coefficient is increased to 10^−3^ to limit the magnitude of parameter updates in a single step. After a small number of training iterations, the residual characteristics of the network degrade toward the target instance with a moderate shift, completing the adaptation.

### 3.4. Overall Framework and Implementation Workflow

**Figure 3:**
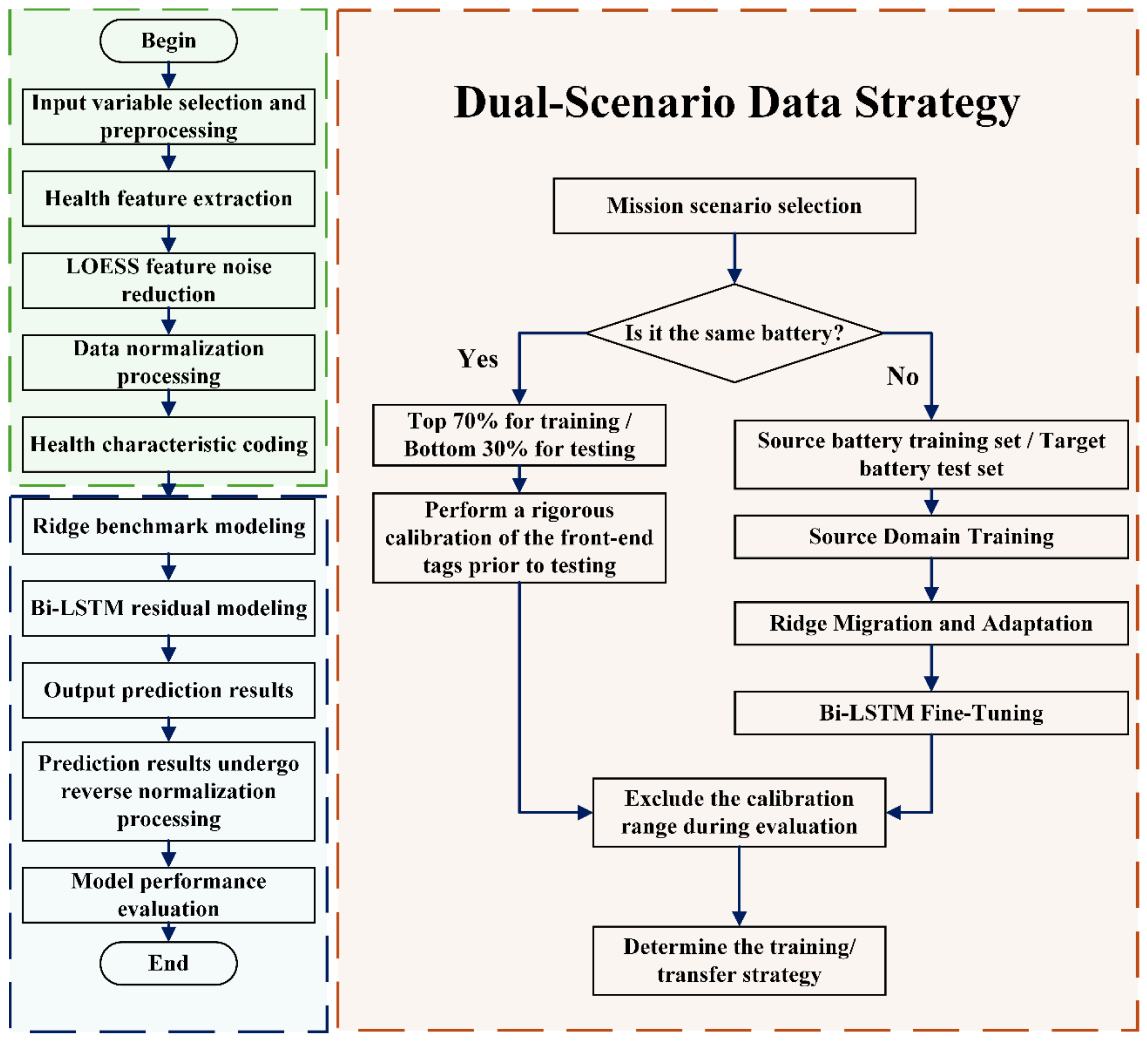
The overall architecture diagram of Ridge-Conv-Bi-LSTM.

The Ridge-Conv-Bi-LSTM framework with dual-level transfer learning first establishes a Ridge regression baseline to capture the global degradation trend, from which a Conv-Bi-LSTM network learns the nonlinear residuals. A dual-level transfer mechanism is employed, wherein the Ridge coefficients are first adapted through prior-regularized regression, followed by lightweight fine-tuning of the pre-trained Conv-Bi-LSTM network, jointly enabling rapid target-battery adaptation with limited calibration data. The overall architecture diagram of Ridge-Conv-Bi-LSTM is shown in Figure 3.

## 4. Experimental Scenarios and Ablation Analysis

### 4.1. Experimental Setup

The proposed Ridge-Conv-Bi-LSTM framework is validated on cycle aging data from batteries B1–B6 (Tsinghua), C1–C3 (Oxford), and D1–D6 (Tongji) under three evaluation scenarios: single-battery extrapolation, cross-battery transfer, and ablation analysis. Performance is compared against Ridge regression, LSTM, Bi-LSTM, and random forest (RF) in terms of estimation accuracy, generalization, and stability.

In the single-battery scenario, the data are chronologically divided into a training segment and a subsequent segment at a 7:3 ratio, with the first 25% of the subsequent segment used for calibration and the remaining 75% for testing. In the cross-battery transfer experiments, the initial cycles of the target domain, determined by an adaptive rule, are used for model calibration, while the subsequent cycles are used for performance evaluation. Specifically, 15% of the total target-domain cycles is first adopted as the nominal calibration sample size; the number of calibration samples is then constrained to between 80 and 180 cycles and is simultaneously limited to no more than 35% of the total target-domain cycles. Consequently, the actual calibration proportion varies adaptively with the size of the target-domain data across different transfer tasks, and the corresponding evaluation proportion is therefore not a fixed value. Core hyperparameters for both scenarios are listed in Table 1 and Table 2.

The hyperparameters in Table 1 and Table 2 were determined through preliminary coarse tuning using only the training and calibration portions of the data; the test portions were not involved in parameter selection. To limit the number of tunable factors, the principal network configuration—32 convolutional filters with a kernel size of 3, 64 Bi-LSTM hidden units, 32 fully connected units, and a dropout rate of 0.12—was kept identical in the two evaluation scenarios. This moderate network size was adopted to provide sufficient nonlinear representation capacity while reducing the risk of overfitting on the relatively small battery datasets. The initial learning rate of $7\times 10^{-4}$ was used for source-model training, whereas the lower learning rate of $2\times 10^{-4}$ was used during target-domain fine-tuning to avoid excessive modification of the transferred parameters. A slightly stronger L2 regularization coefficient was therefore used during fine-tuning because substantially fewer target-domain samples were available. The sliding-window length, mini-batch size, and maximum number of epochs were adjusted only between the single-battery and cross-battery scenarios to account for their different sequence lengths and sample sizes. Once selected, all hyperparameters were fixed for every battery within the corresponding scenario and were not reoptimized on the test sets.

Additionally, this paper selects RMSE, mean absolute error (MAE), mean absolute percentage error (MAPE), and coefficient of determination (R^2^) as evaluation metrics, with the corresponding formulas as follows [33].

(9) $$RMSE=\sqrt{\frac{1}{N}\sum_{i=1}^{N}{\left(\hat{y_{i}}-y_{i}\right)}^{2}}$$

(10) $$MAE=\frac{1}{N}\sum_{i=1}^{N}\left|\hat{y_{i}}-y_{i}\right|$$

(11) $$MAPE=\frac{1}{N}\sum_{i=1}^{N}\frac{\left|\hat{y_{i}}-y_{i}\right|}{y_{i}}\times 100\%$$

(12) $$R^{2}=1-\frac{\sum_{i=1}^{N}{\left(\hat{y_{i}}-y_{i}\right)}^{2}}{\sum_{i=1}^{N}{\left(y_{i}-\bar{y}\right)}^{2}}$$

Within the above Equation, $y_{i}$ is the true value, $\hat{y_{i}}$ is the estimated value, $\bar{y}$ is the mean of the true value, where *N* is the sample size.

### 4.2. Single-Battery Extrapolation

Six cells were selected for the single-battery extrapolation scenario: B1, B4, and B6 from Tsinghua, and D2, D4, and D6 from Tongji. The proposed Ridge-Conv-Bi-LSTM and the four baseline methods were each run 20 times independently, with the final predictions obtained by averaging across all runs.

**Figure 4:**
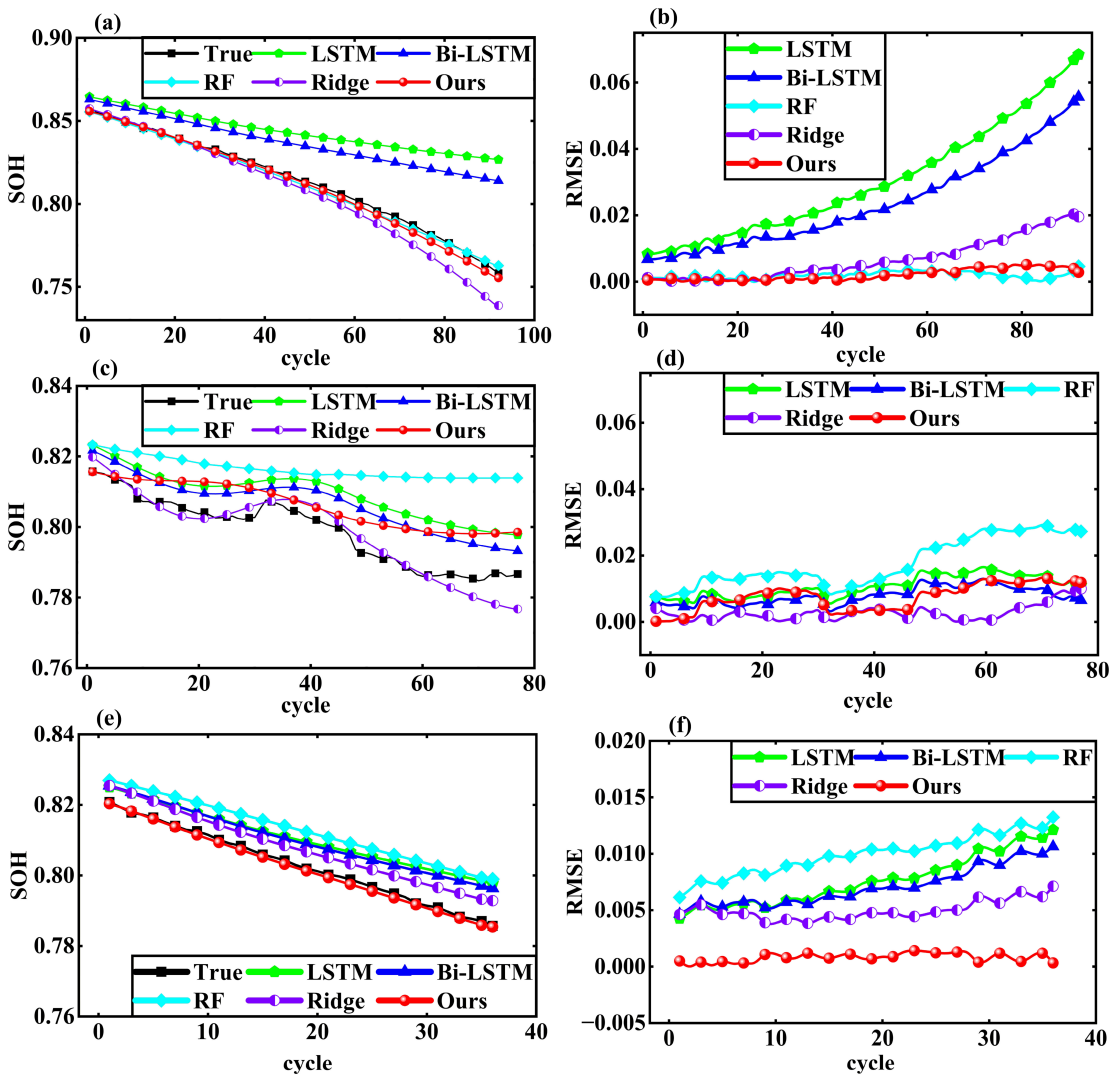
Comparison of battery SOH estimation results from different models in the Tsinghua University dataset across single-battery extrapolation: (**a**) B1 battery SOH estimation; (**b**) B1 battery SOH estimation error; (**c**) B4 battery SOH estimation; (**d**) B4 battery SOH estimation error; (**e**) B6 battery SOH estimation; (**f**) B6 battery SOH estimation error.

Figure 4 shows the comparison between the predicted and actual battery SOH values on the Tsinghua University dataset. The black curve represents the true SOH values, while the red curve denotes the estimation results obtained by the proposed method. The green, blue, cyan, and purple curves correspond to the prediction results of the LSTM, Bi-LSTM, RF, and Ridge models, respectively.

As shown in Figure 4a,c,e, the proposed method generally provides SOH predictions that closely follow the actual degradation trajectories. Compared with the LSTM, Bi-LSTM, RF, and Ridge models, the proposed method demonstrates better agreement with the true SOH values for most operating cycles. Although larger deviations can be observed in certain regions, particularly in Figure 4c, the proposed method is still capable of effectively capturing the overall degradation trend of battery SOH.

The RMSE results in Figure 4b,d,f further illustrate prediction performance. Although not always yielding the lowest error at every point, the proposed method generally maintains more stable RMSE values than the compared models, confirming its reliable SOH estimation across the room-temperature, moderate-temperature, and high-temperature conditions of 25 °C, 35 °C, and 45 °C.

**Figure 5:**
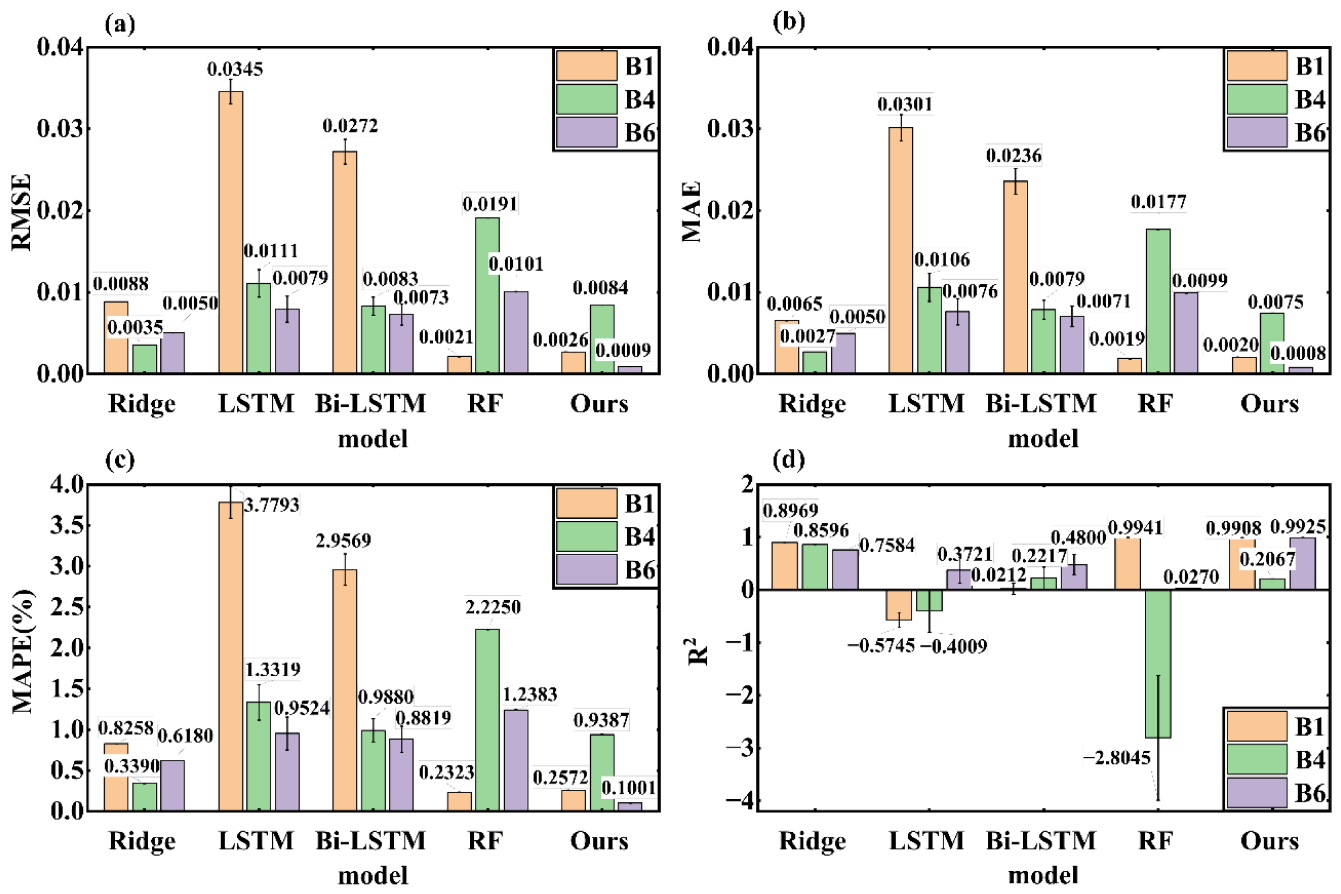
Evaluation metrics for SOH estimation across Tsinghua University dataset in single-battery extrapolation situation: (**a**) RMSE; (**b**) MAE; (**c**) MAPE; (**d**) R^2^.

The various errors—RMSE, MAE, MAPE, R^2^—estimated by each algorithm across Tsinghua University dataset are compared in Figure 5.

As shown in Figure 5a, the proposed method achieves the lowest RMSE value of 0.0009 for battery B6, indicating the highest prediction accuracy among all compared models. The RMSE values obtained by Ridge, LSTM, Bi-LSTM, and RF are 0.0050, 0.0079, 0.0073, and 0.0101, respectively. Compared with these methods, the proposed approach reduces RMSE by 82.0% (vs. Ridge), 88.6% (vs. LSTM), 87.7% (vs. Bi-LSTM), and 91.1% (vs. RF). Among all baseline models, the largest improvement is observed over LSTM, followed by Bi-LSTM, Ridge, and RF. As illustrated in Figure 5b–d, the proposed method also achieves competitive performance in terms of MAE, MAPE, and R^2^. Although it does not consistently obtain the best value for every evaluation metric and battery, the overall results demonstrate lower prediction errors and better fitting performance compared with benchmark models.

**Figure 6:**
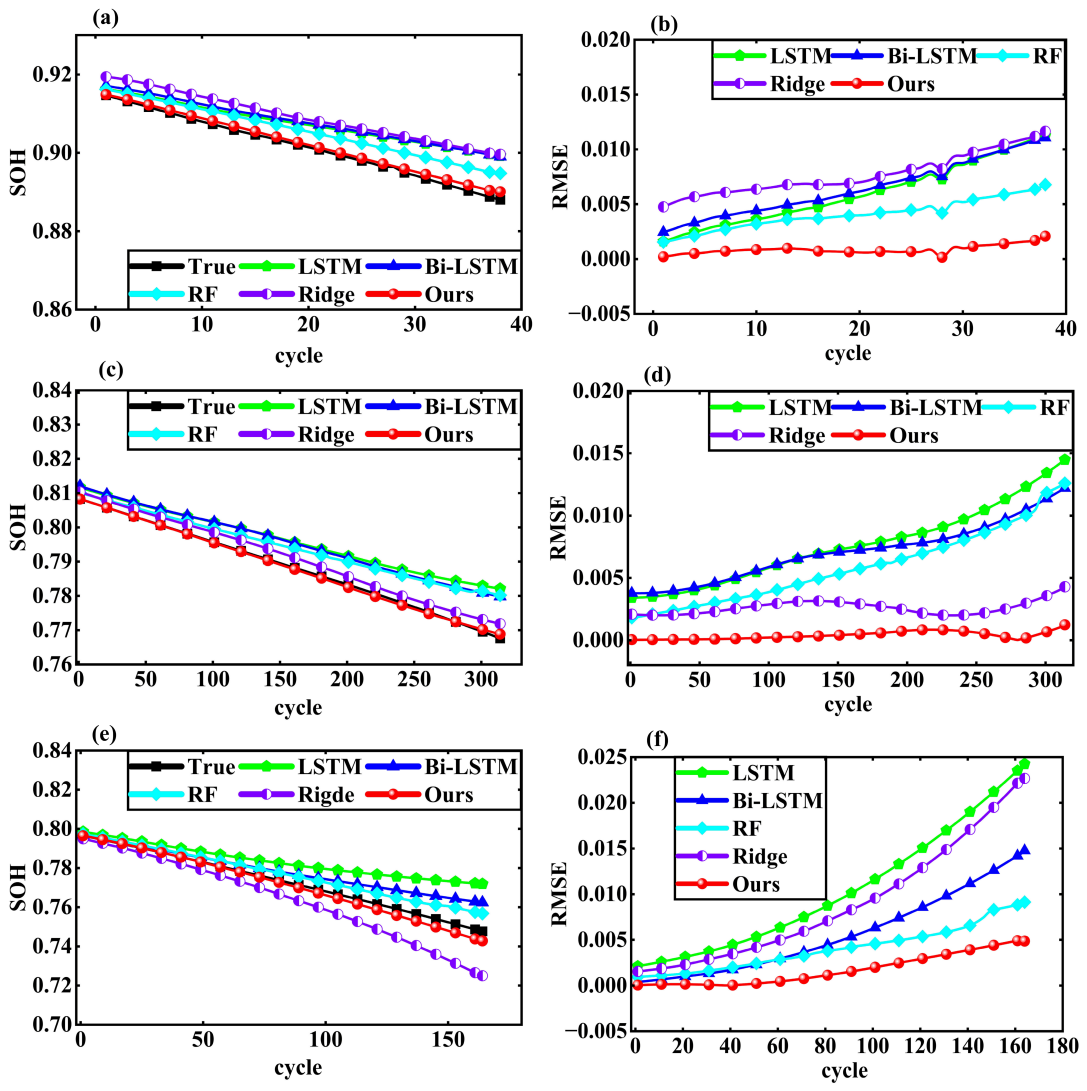
Comparison of battery SOH estimation results from different models in the Tongji University dataset across single-battery extrapolation: (**a**) D2 battery SOH estimation; (**b**) D2 battery SOH estimation error; (**c**) D4 battery SOH estimation; (**d**) D4 battery SOH estimation error; (**e**) D6 battery SOH estimation; (**f**) D6 battery SOH estimation error.

Figure 6 shows the comparison between the predicted and actual battery SOH values on the Tongji University dataset.

As shown in Figure 6a,c,e, the proposed method accurately tracks the SOH degradation trends of the Tongji University dataset under different temperature conditions, including 25 °C, 35 °C, and 45 °C. Compared with LSTM, Bi-LSTM, RF, and Ridge, the proposed method shows better agreement with the measured SOH values. Although slight deviations can be observed in some regions, particularly in Figure 6e, the overall degradation behavior is still well captured.

The RMSE results in Figure 6b,d,f further demonstrate the prediction performance of the proposed method. Under both room-temperature and high-temperature conditions, the proposed method generally achieves lower and more stable errors than the baseline models throughout the cycling process. These results indicate that the proposed algorithm can provide accurate and robust SOH estimation under varying thermal conditions.

**Figure 7:**
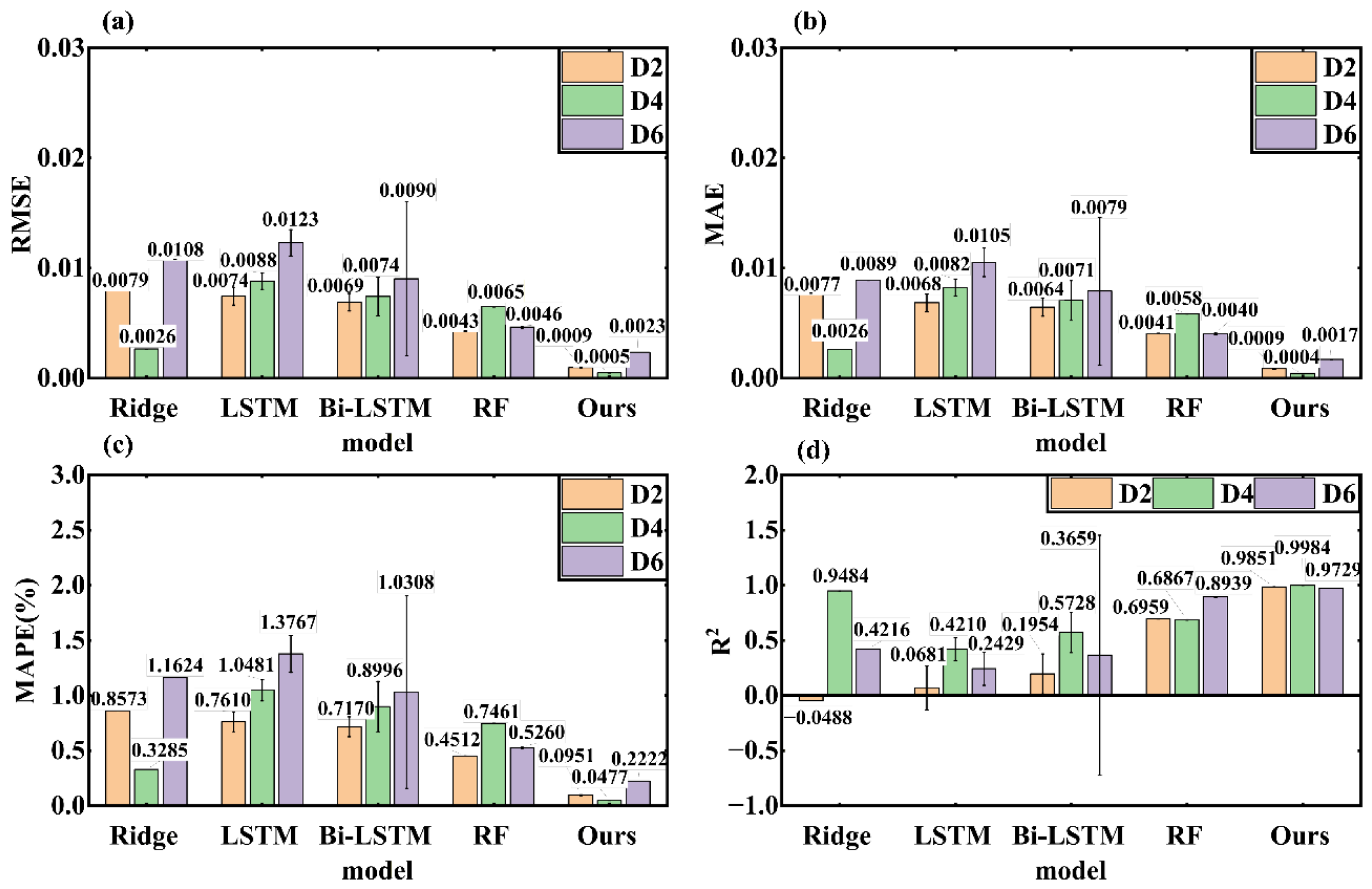
Evaluation metrics for SOH estimation across Tongji University dataset in single-battery extrapolation situation: (**a**) RMSE; (**b**) MAE; (**c**) MAPE; (**d**) R^2^.

The various errors—RMSE, MAE, MAPE, R^2^—estimated by each algorithm across Tongji University dataset are compared in Figure 7.

Figure 7 compares the prediction performance of different models on the D2, D4, and D6 batteries. For the D6 battery under the high-temperature condition of 45 °C, the proposed method achieves an RMSE of 0.0023, which is significantly lower than those of Ridge, LSTM, Bi-LSTM, and RF. Specifically, the RMSE is reduced by 78.7%, 81.3%, 74.4%, and 50.0% compared with Ridge, LSTM, Bi-LSTM, and RF, respectively. This result indicates that the method can maintain high SOH estimation accuracy under high-temperature degradation conditions.

In addition, the results of MAE, MAPE, and R^2^ further demonstrate the superiority of the proposed method. Compared with the baseline models, the proposed method achieves lower MAE and MAPE values and higher R^2^ values across the evaluated batteries, confirming its improved accuracy and robustness for SOH estimation under different operating temperatures.

### 4.3. Cross-Battery Transfer

Cross-battery transfer experiments were performed on three datasets. For Tsinghua cells, the source–target pairs were B1→B2, B4→B3, and B5→B6. For Oxford cells, C1→C2 and C2→C3 were evaluated. For Tongji cells, D1→D2, D3→D4, and D5→D6 were tested. In each pair, the source cell data were used for pre-training, and the target cell provided limited calibration data for transfer adaptation. All models were run 20 times independently, and the ensemble-averaged predictions were used for metric calculation.

**Figure 8:**
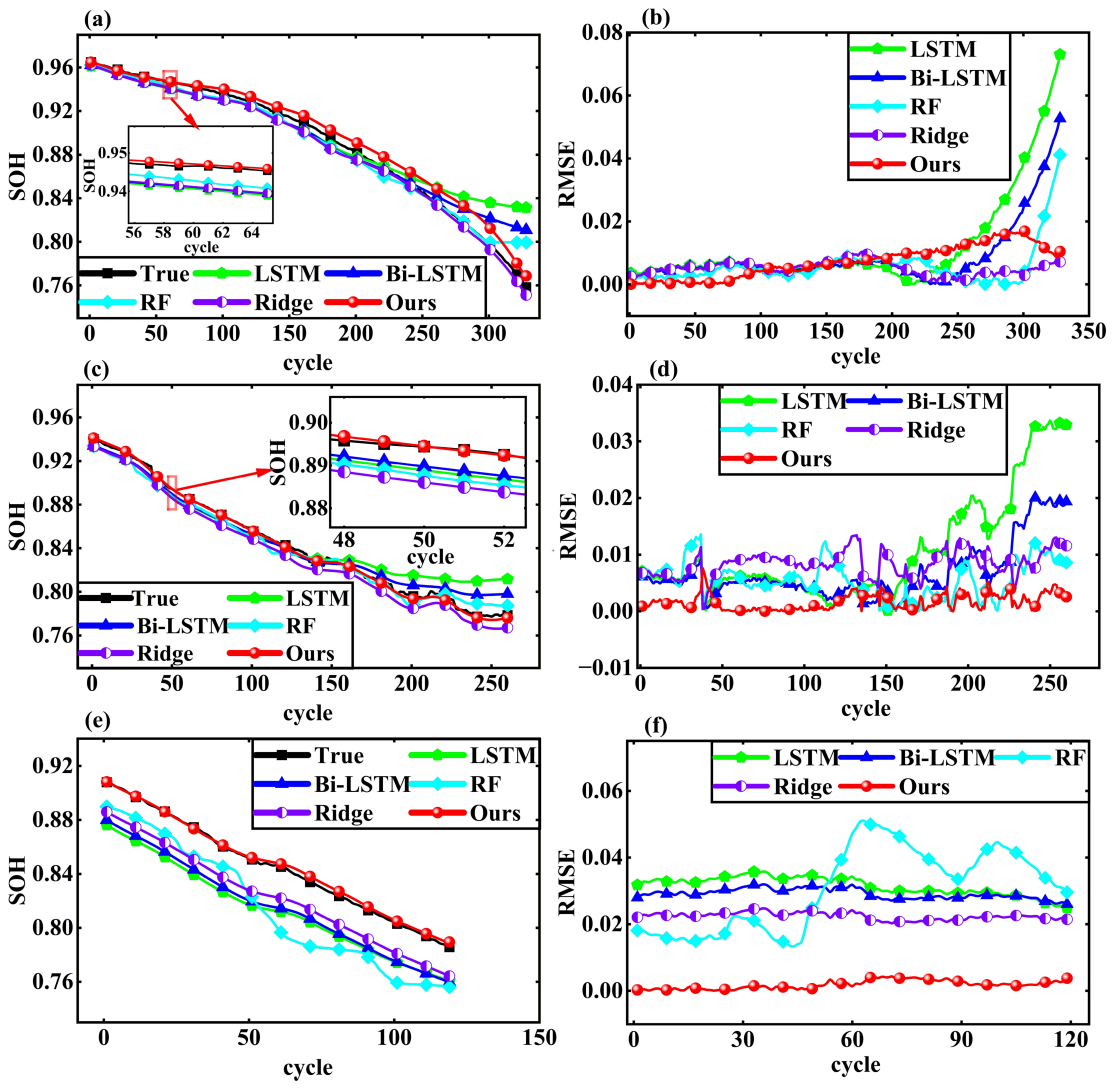
Comparison of battery SOH estimation results from different models in the Tsinghua University dataset across battery transfer situation: (**a**) B2 battery SOH estimation; (**b**) B2 battery SOH estimation error; (**c**) B4 battery SOH estimation; (**d**) B4 battery SOH estimation error; (**e**) B6 battery SOH estimation; (**f**) B6 battery SOH estimation error.

Figure 8 shows the comparison between the predicted and actual battery SOH values on the Tsinghua University dataset.

Overall, the predicted SOH curves and RMSE results show that the proposed dual-transfer-learning-based Ridge-Conv-Bi-LSTM method performs well in cross-battery SOH estimation. From a macroscopic perspective, the estimated SOH values closely follow the measured degradation trajectories in Figure 8a,c,e while the RMSE curves in Figure 8b,d,f remain relatively low and stable over most cycles.

From a microscopic perspective, the proposed method also shows better local stability than the comparison models. Taking Figure 8d as an example, the RMSE curve of the proposed method exhibits smaller fluctuations during cycling, whereas LSTM, Bi-LSTM, RF, and Ridge show more pronounced error variations in several regions. This suggests that the proposed framework can reduce local prediction uncertainty and improve robustness. Therefore, the dual-transfer-learning-based Ridge-Conv-Bi-LSTM has clear advantages in cross-battery SOH estimation, especially in terms of accuracy, stability, and generalization capability.

**Figure 9:**
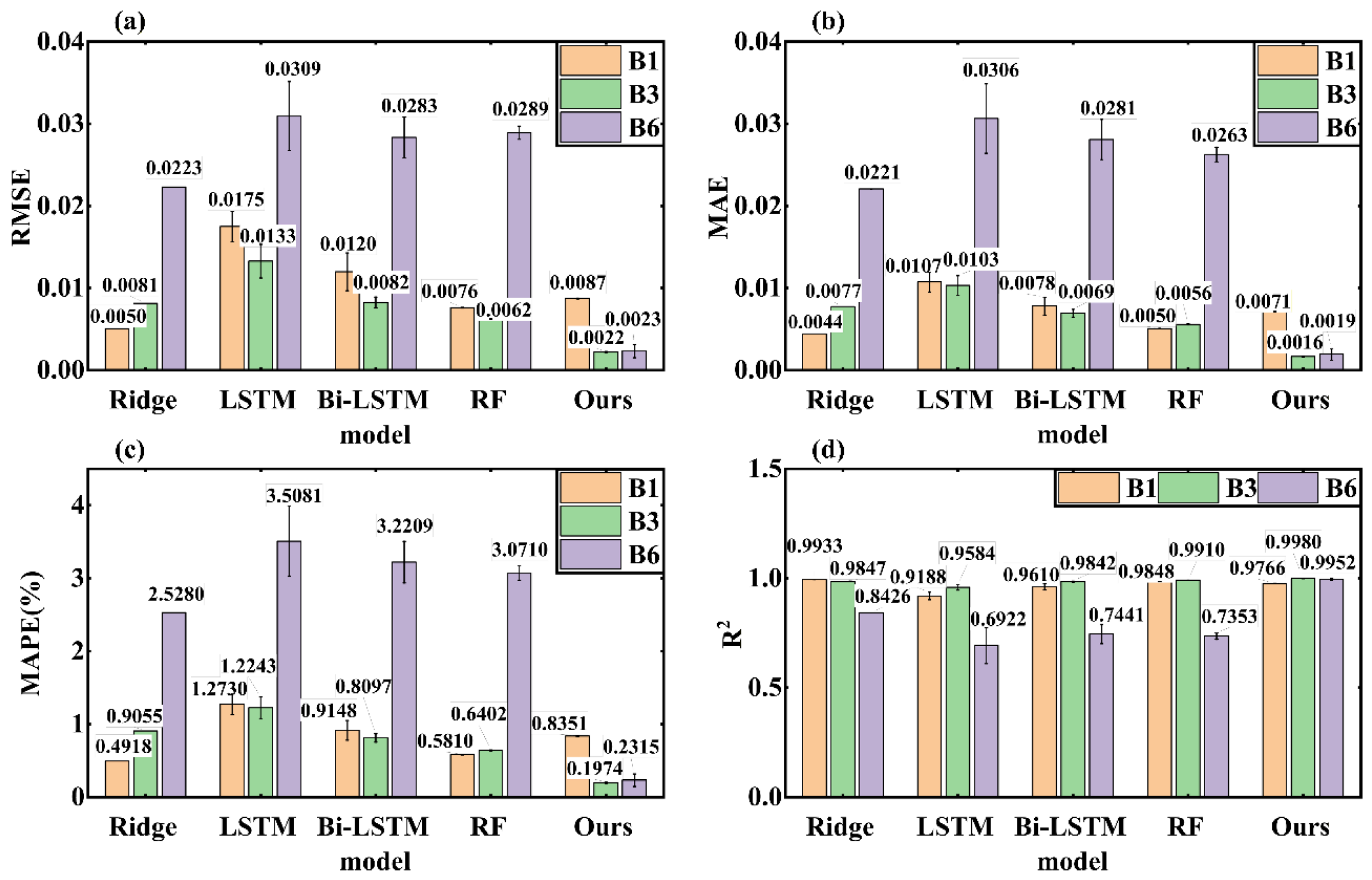
Evaluation metrics for SOH estimation across Tsinghua University dataset in battery transfer situation: (**a**) RMSE; (**b**) MAE; (**c**) MAPE; (**d**) R^2^.

Figure 9 compares the RMSE, MAE, MAPE, and R^2^ values obtained by different algorithms on the Tsinghua University dataset.

As illustrated in Figure 9, the proposed method exhibits more stable and competitive performance across RMSE, MAE, MAPE, and R^2^ compared with Ridge, LSTM, Bi-LSTM, and RF. Although the estimation performance on the B1 battery is not locally optimal in some metrics, the proposed method achieves a better overall balance among prediction accuracy, error stability, and fitting capability. In particular, for the B6 battery under the 50 °C high-temperature condition, the proposed method obtains an RMSE of 0.0023, which is much lower than those of Ridge, LSTM, Bi-LSTM, and RF, with corresponding reductions of 89.7%, 92.6%, 91.9%, and 92.0%, respectively. These results indicate that the proposed method maintains high SOH estimation accuracy and strong robustness under high-temperature battery degradation conditions.

**Figure 10:**
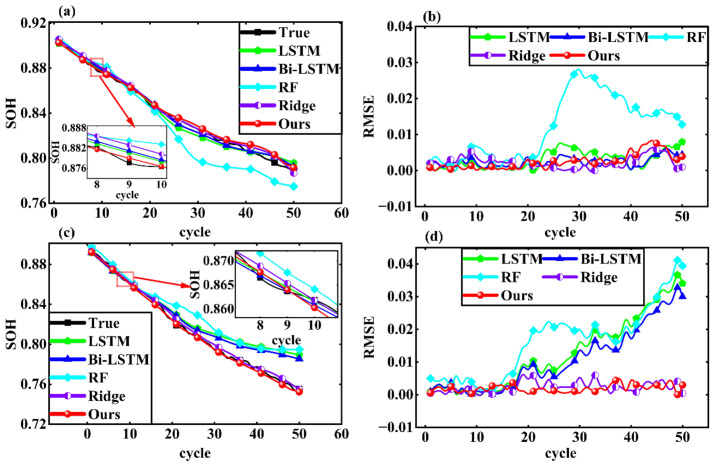
Comparison of battery SOH estimation results from different models in the University of Oxford dataset across battery transfer situation: (**a**) C2 battery SOH estimation; (**b**) C2 battery SOH estimation error; (**c**) C3 battery SOH estimation; (**d**) C3 battery SOH estimation error.

Figure 10 presents the comparison between the estimated and measured battery SOH values on the University of Oxford dataset.

Figure 10a,c show that the proposed method captures the overall battery SOH degradation trend, and its estimated curves are closer to the measured values than those from LSTM, Bi-LSTM, RF, and Ridge, indicating better tracking and prediction accuracy under different aging conditions. Figure 10b,d show that the proposed method yields a low and stable RMSE over most cycles, whereas the comparison models exhibit larger fluctuations, especially in later cycles, confirming its improved SOH estimation reliability, accuracy, and robustness.

**Figure 11:**
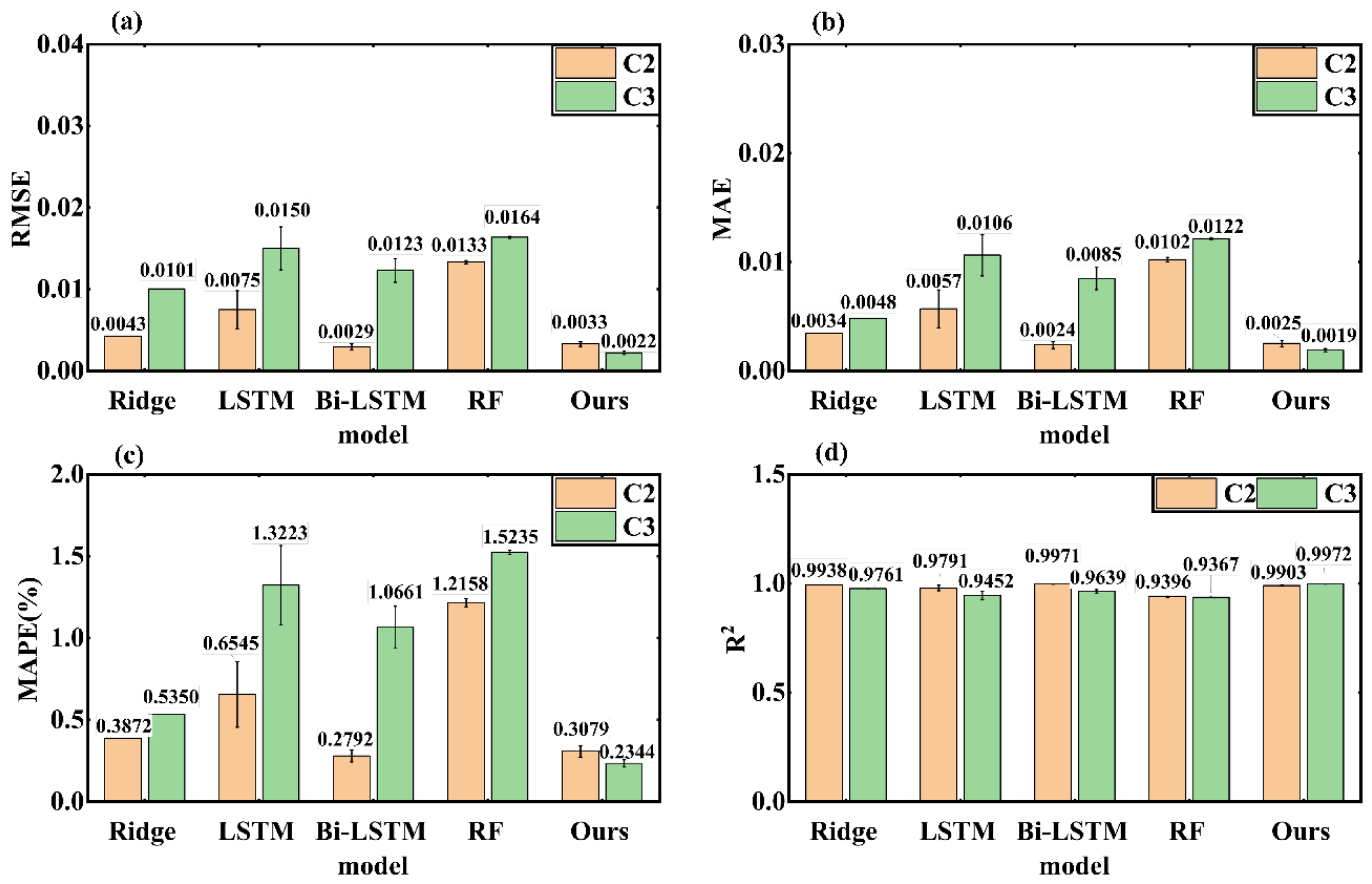
Evaluation metrics for SOH estimation across the University of Oxford dataset in battery transfer situation: (**a**) RMSE; (**b**) MAE; (**c**) MAPE; (**d**) R^2^.

Figure 11 compares the RMSE, MAE, MAPE, and R^2^ values obtained by different algorithms on the University of Oxford dataset.

Figure 11 presents that the proposed method achieves competitive SOH estimation performance on the C2 and C3 batteries, with relatively low errors and high fitting accuracy across RMSE, MAE, MAPE, and R^2^. Focusing on the RMSE results of the C2 battery, the proposed method obtains an RMSE of 0.0033. Compared with Ridge, LSTM, and RF, the RMSE is reduced by 23.3%, 56.0%, and 75.2%, respectively. Although the RMSE is slightly higher than that of Bi-LSTM on C2, the proposed method still shows better overall performance when considering multiple evaluation metrics and different batteries.

**Figure 12:**
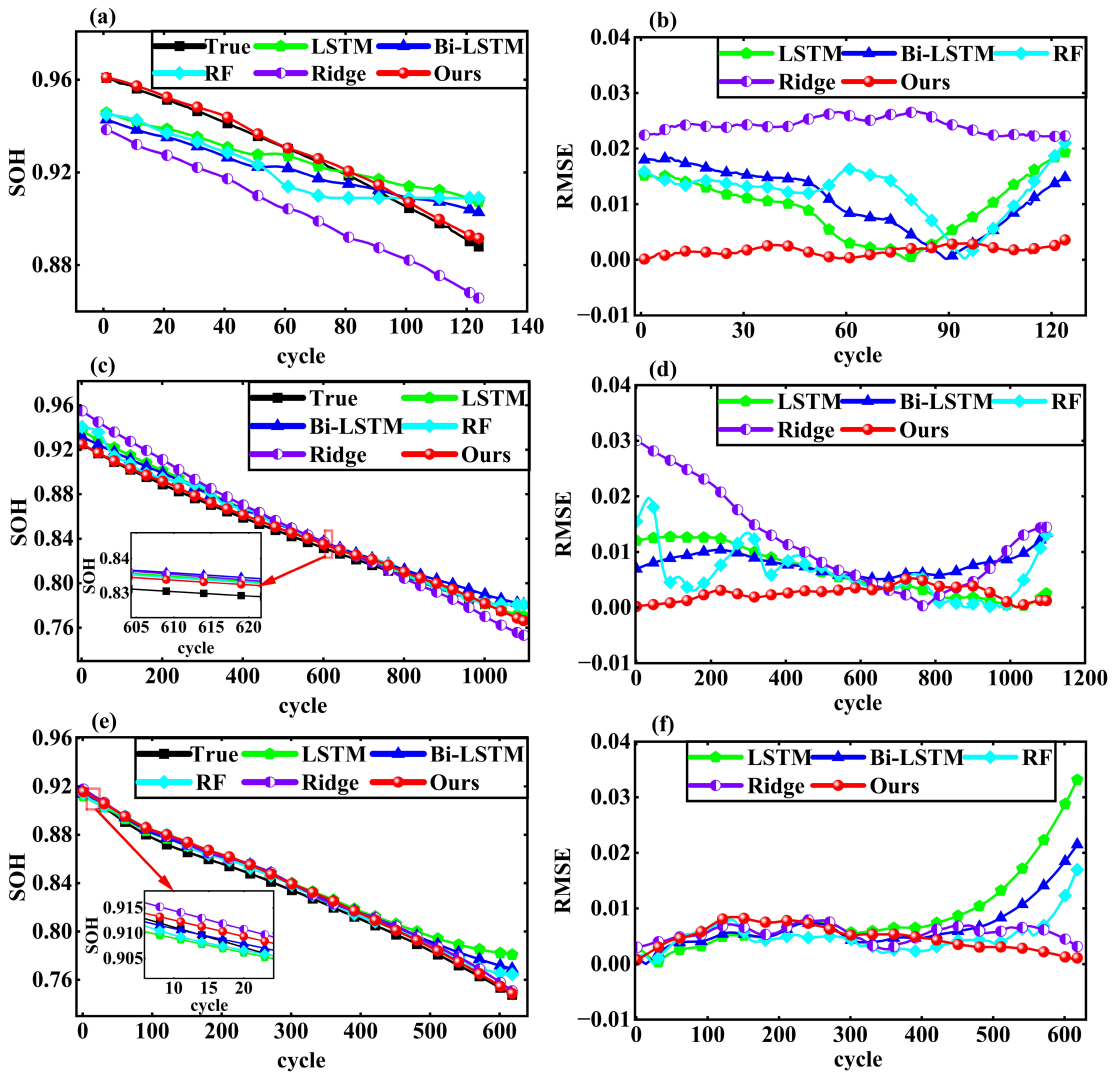
Comparison of battery SOH estimation results from different models in the Tongji University dataset across battery transfer situation: (**a**) D2 battery SOH estimation; (**b**) D2 battery SOH estimation error; (**c**) D4 battery SOH estimation; (**d**) D4 battery SOH estimation error; (**e**) D6 battery SOH estimation; (**f**) D6 battery SOH estimation error.

Figure 12 illustrates the agreement between the estimated and measured battery SOH values on the Tongji University dataset.

Figure 12 illustrates the battery SOH estimation performance under different temperature conditions. Focusing on the last two subplots, corresponding to the 45 °C high-temperature condition, the proposed method accurately tracks the SOH degradation trend. While the prediction performance of the proposed method and Ridge is similar in the early and middle cycles, the proposed method exhibits superior accuracy towards the end of the cycling process. These results indicate that the proposed algorithm maintains high estimation accuracy not only at room temperature (25 °C) and moderate-high temperature (35 °C) but also under high-temperature conditions (45 °C), demonstrating its robustness across different thermal environments.

**Figure 13:**
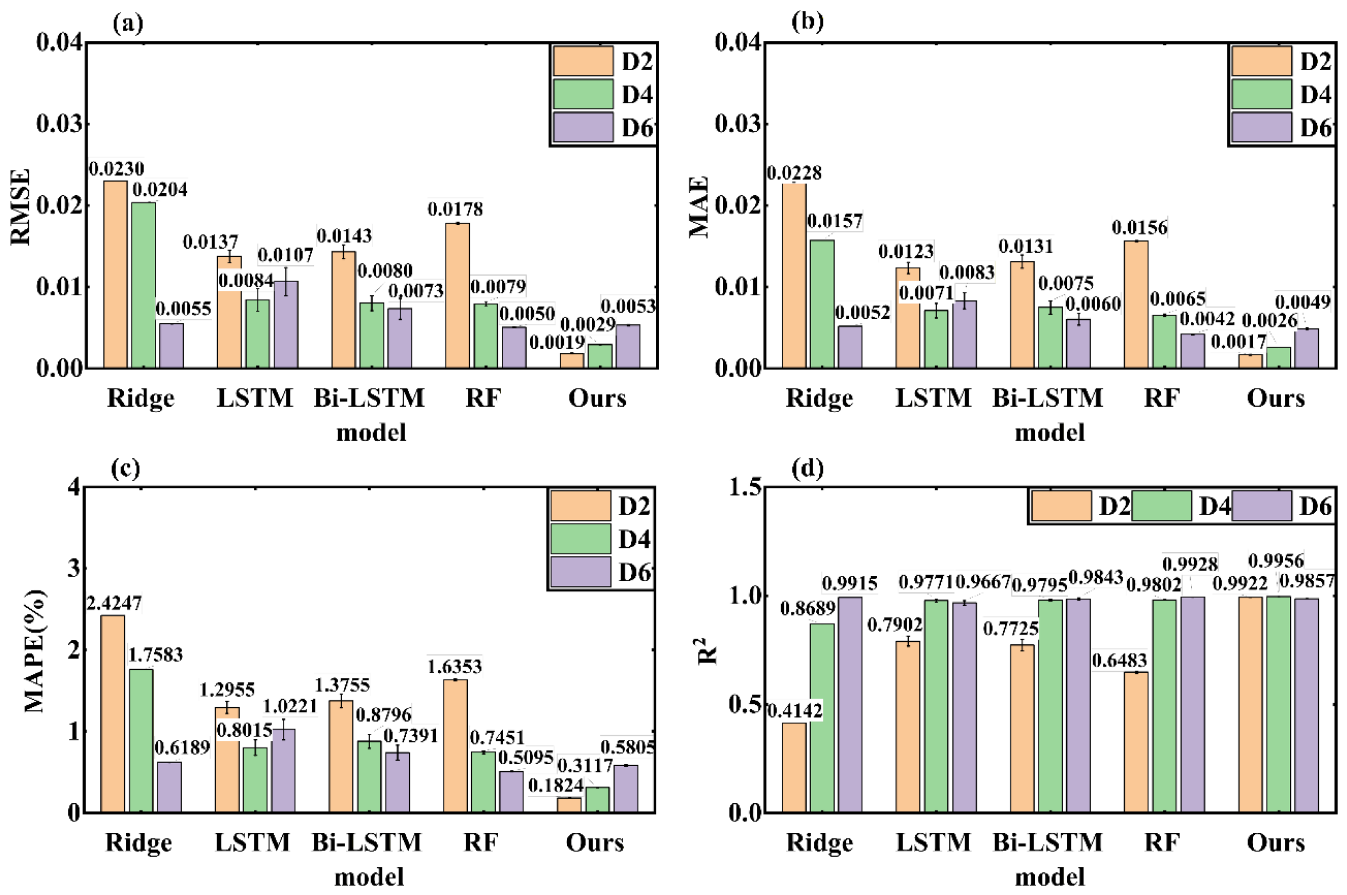
Evaluation metrics for SOH estimation across the Tongji University dataset in battery transfer situation: (**a**) RMSE; (**b**) MAE; (**c**) MAPE; (**d**) R^2^.

Figure 13 presents a comparative evaluation of RMSE, MAE, MAPE, and R^2^ for different algorithms on the Tongji University dataset.

Figure 13 indicates that the proposed model achieves the best overall performance in the cross-battery SOH estimation task on the Tongji University dataset, though it is not the best for every single metric. Focusing on the D4 battery under the 35 °C condition, the proposed method achieves an RMSE of approximately 0.0029, corresponding to an estimation accuracy improvement of 85.8%, 65.5%, 63.3%, and 63.8% over Ridge, LSTM, RF, and Bi-LSTM, respectively. These results highlight the advantage of the proposed method for battery SOH estimation under medium-high temperature conditions, demonstrating both improved accuracy and robustness across different batteries.

### 4.4. Ablation Analysis

**Figure 14:**
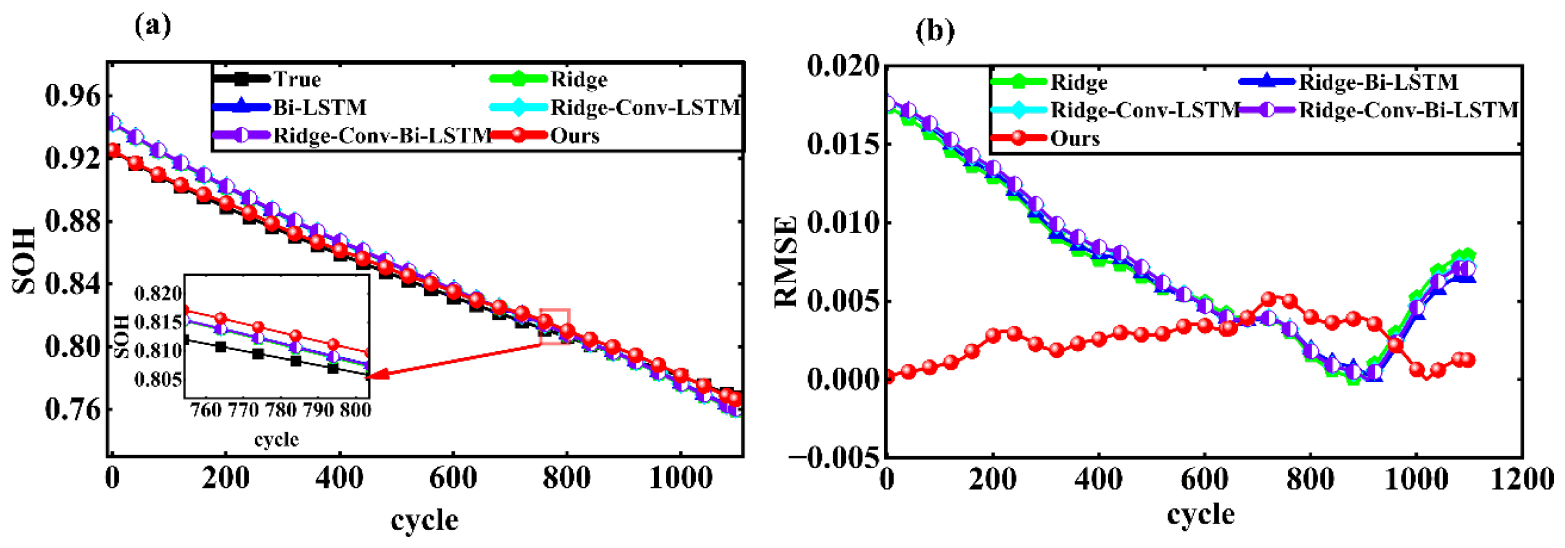
Comparison of SOH estimation results for the D4→D3 cross-battery task on the Tongji University dataset: (**a**) estimated and measured SOH of the test cell D3; (**b**) SOH estimation error for the test cell D3.

Figure 14 compares the estimated and true battery SOH values for the ablation experiment on the Tongji University dataset, where cell D4 was used for training and cell D3 for testing. The predicted curves represent the ensemble averages over 20 independent runs for each model variant.

As shown in Figure 14, the ablation results compare the estimated and measured SOH values under different model configurations. The four baseline models exhibit similar degradation-tracking trends and error patterns, with relatively large deviations in the early and late cycling stages. In contrast, the proposed method based on dual transfer learning achieves a closer agreement with the measured SOH trajectory and maintains lower RMSE values throughout most cycles. This indicates that the performance improvement is not simply obtained by stacking individual models, since the direct combination of single models does not effectively enhance SOH estimation accuracy. Instead, the dual transfer learning strategy plays a vital role in improving prediction accuracy and robustness.

**Figure 15:**
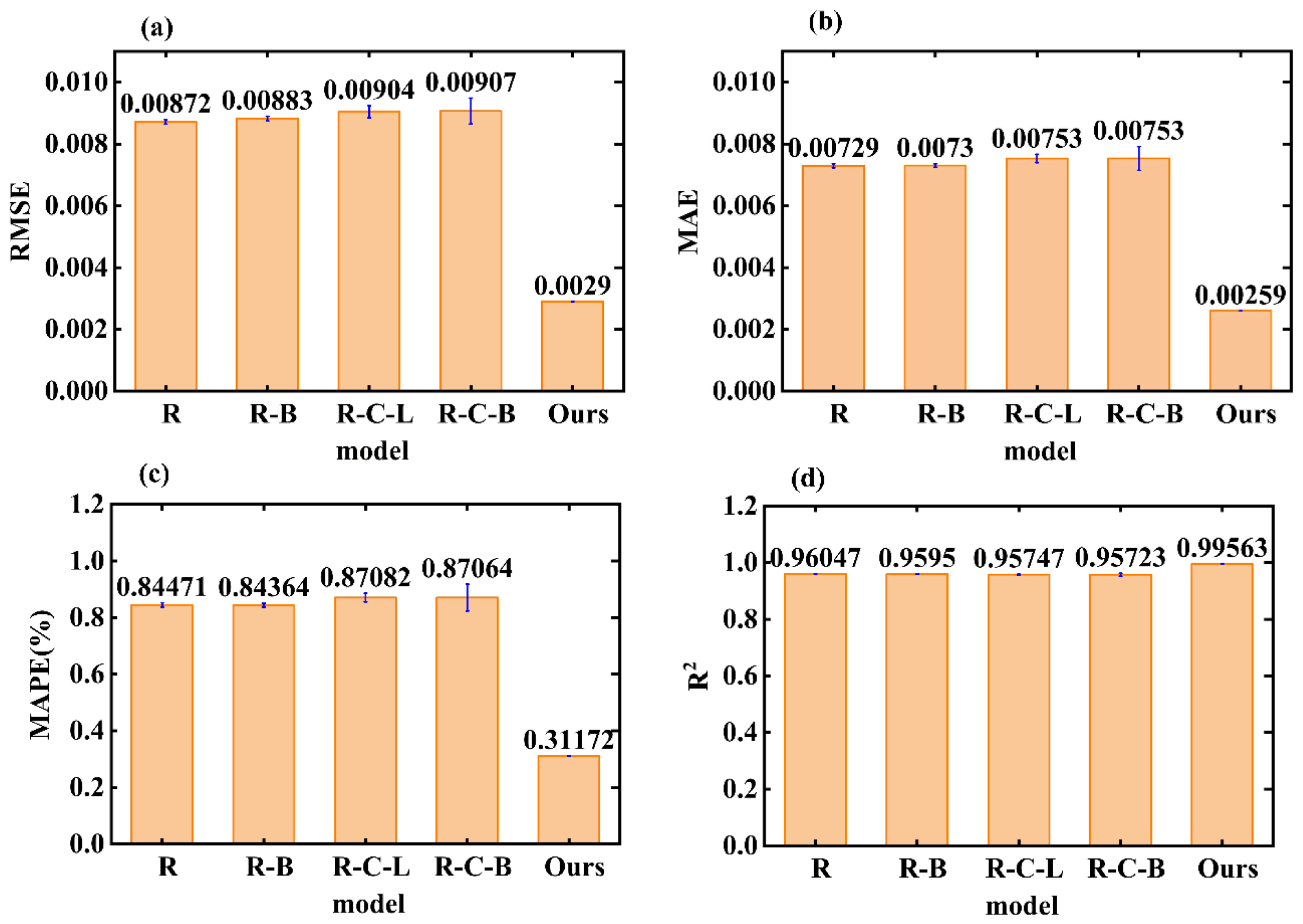
Evaluation metrics for SOH estimation across the Tongji University dataset: (**a**) RMSE; (**b**) MAE; (**c**) MAPE; (**d**) R^2^. (R: Ridge, R-B: Ridge-Bi-LSTM; R-C-L: Ridge-Conv-LSTM; R-C-B: Ridge-Conv-Bi-LSTM.).

Figure 15 presents a comparative evaluation of RMSE, MAE, MAPE, and R^2^ for different algorithms on the D3 battery.

Figure 15 distinguishes the contribution of model architecture from that of transfer adaptation. Among the four variants without target-domain adaptation, increasing the model complexity from Ridge to Ridge–Conv–Bi-LSTM does not improve cross-battery generalization. Specifically, the RMSE increases from 0.00872 to 0.00907, the MAE increases from 0.00729 to 0.00753, and the MAPE increases from 0.84471% to 0.87064%, while R^2^ decreases from 0.96047 to 0.95723. These results indicate that merely introducing convolutional and recurrent layers cannot effectively compensate for the distribution discrepancy between the source and target batteries. In contrast, after incorporating the proposed dual-level transfer learning strategy into the same Ridge–Conv–Bi-LSTM backbone, the RMSE, MAE, and MAPE decrease to 0.00290, 0.00259, and 0.31172%, respectively. Compared with the non-transfer Ridge–Conv–Bi-LSTM model, these values correspond to reductions of 68.0%, 65.6%, and 64.2%, respectively. Meanwhile, R^2^ increases from 0.95723 to 0.99563, corresponding to an approximately 89.8% reduction in the unexplained variance. Moreover, the performance gaps between the complete model and the non-transfer variants are substantially larger than the standard deviations obtained from the repeated experiments, indicating that the improvements are robust to random initialization. Therefore, under the investigated cross-battery setting, the main performance gain arises from the proposed transfer adaptation mechanism rather than from simply increasing the structural complexity of the prediction network.

**Figure 16:**
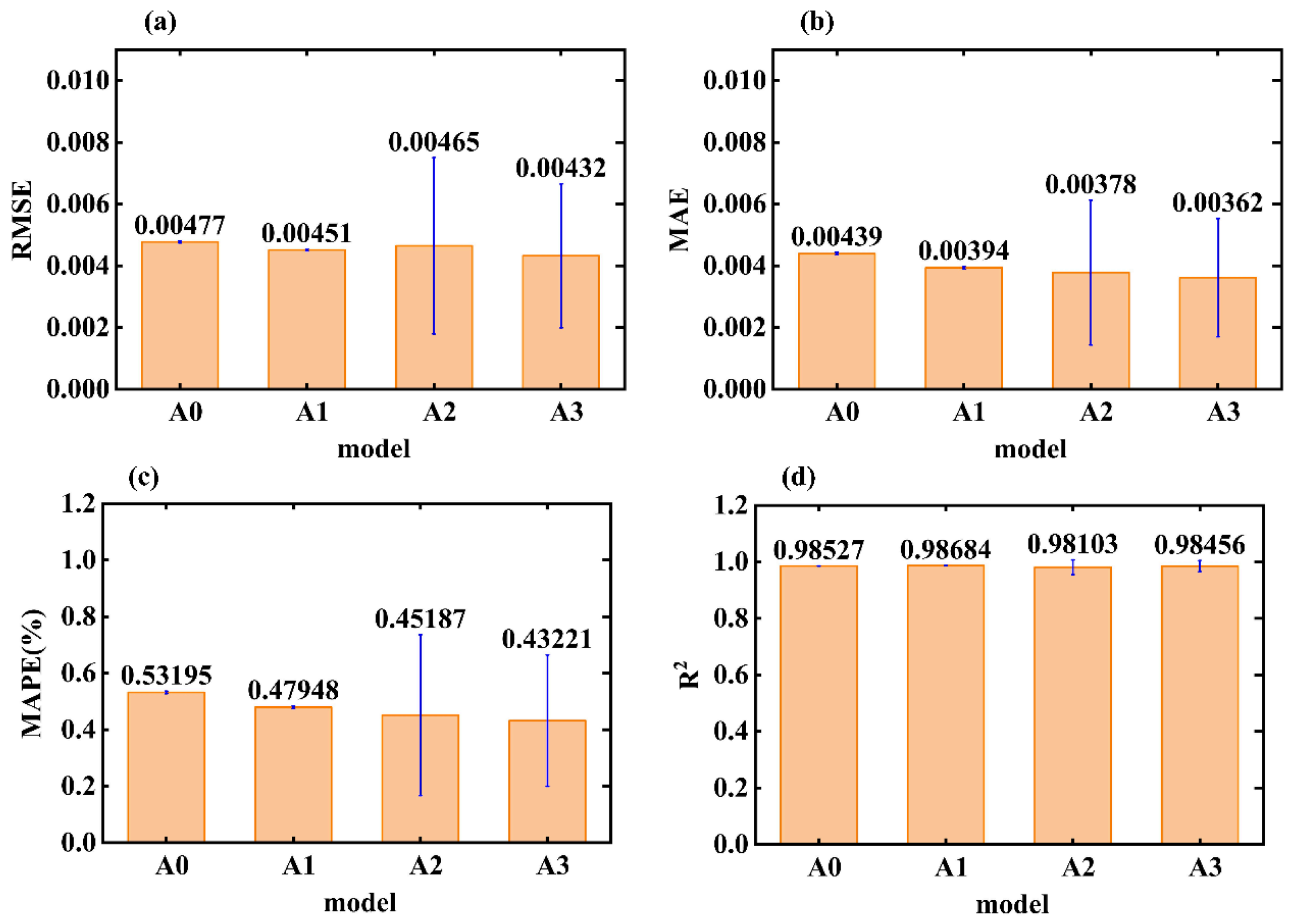
Hierarchical ablation of the dual-level transfer strategy for the D4→D3 task with a 15% target-domain calibration proportion: (**a**) RMSE; (**b**) MAE; (**c**) MAPE; and (**d**) R^2^. A0: no target-domain adaptation; A1: Ridge-prior adaptation only; A2: Conv–Bi-LSTM residual-network fine-tuning only; A3: complete dual-level adaptation. Bars and error bars represent the mean and ± one standard deviation over 20 independent runs, respectively.

To further isolate the contributions of different adaptation levels within the dual-level transfer learning strategy, a hierarchical ablation experiment was conducted using battery D4 from Tongji University as the source domain and battery D3 as the target domain, with the target-domain calibration proportion fixed at 15%. Four configurations, denoted as A0–A3, were established: A0, without any target-domain adaptation; A1, with only Ridge prior coefficient adaptation; A2, with only Conv–Bi-LSTM residual network fine-tuning; and A3, with the complete dual-level transfer learning strategy combining both adaptation mechanisms. All configurations employed identical data splits, network architectures, and training hyperparameters and were independently evaluated using the same 20 random seeds. The corresponding results are presented as bar charts with error bars in Figure 16.

A1 (Ridge prior adaptation only) reduced RMSE, MAE, and MAPE by 5.48%, 10.42%, and 9.86%, respectively, compared with A0, and raised the mean R^2^ from 0.985268 to 0.986839. The consistently small standard deviations indicate that constraining the global degradation prior with early target-domain samples stably reduces the trend discrepancy between source and target batteries.

A2 (Conv–Bi-LSTM residual network fine-tuning only) reduced RMSE, MAE, and MAPE by 2.65%, 13.99%, and 15.05% relative to A0, but its RMSE standard deviation (0.002855) was markedly higher, reflecting the sensitivity of few-shot deep residual network fine-tuning to initialization and optimization.

A3 (complete dual-level transfer) achieved the lowest mean RMSE, MAE, and MAPE (0.004325, 0.003616, and 0.432206%, respectively). These correspond to reductions of 9.39%, 17.70%, and 18.75% over A0; 4.14%, 8.13%, and 9.86% over A1; and 6.92%, 4.32%, and 4.35% over A2. Under identical random seeds, A3 achieved the lowest RMSE among the four configurations in 14 out of 20 independent runs. Although the remaining runs showed slight variations caused by stochastic optimization, A3 consistently achieved the best average performance, demonstrating the complementary contribution of the two transfer stages.

The hierarchical ablation results demonstrate that Ridge prior adaptation and Conv–Bi-LSTM fine-tuning fulfill distinct and complementary functions. The first stage stably corrects the global degradation trend bias across batteries, while the second stage learns battery-specific local nonlinear degradation patterns, further reducing MAE and MAPE at the cost of higher sensitivity to initialization and few-shot optimization. Their joint adoption yields the lowest average errors, validating the necessity of adapting both the global degradation prior and the nonlinear temporal residual representation.

### 4.5. Target-Domain Proportion Calibration Analysis

To investigate the impact of target-domain calibration sample size on transfer prediction performance, this paper presents a sensitivity experiment on target-domain calibration proportions. Eight cross-battery transfer tasks from three datasets are selected, with calibration proportions set to 5%, 10%, 15%, 20%, and 30%. The first 30% of the target battery’s cycles constitute the candidate calibration region, while the remaining 70% serve as the shared test region for all proportions, ensuring identical evaluation samples across different calibration ratios. For each transfer task and calibration proportion, 20 independent runs with different random seeds are performed, and the mean values of RMSE, MAE, MAPE, and R^2^ are reported. The SOH estimation performance of the cross-battery transfer tasks at their optimal target-domain calibration proportions is shown in Table 3.

Table 3 shows that the optimal target-domain calibration proportion is task-dependent rather than fixed. The D1→D2 task reaches its best performance with only 5% of the target data, C1→C2 with 10%, and B2→B1 and B4→B3 with 20%. The remaining four tasks—B5→B6, C2→C3, D3→D4, and D5→D6—require 30%. Thus, although half of the tasks benefit from the largest tested calibration set, several transfers attain their optimum with substantially fewer target-domain samples, indicating that the amount of calibration data required depends on the specific source–target pair.

Across the eight transfer tasks, RMSE ranges from 0.001412 to 0.016004. C1→C2 achieves the lowest RMSE (0.001412), with an MAE reported as 0.000000 at the displayed precision, whereas D1→D2 has the highest RMSE (0.016004) and the lowest R^2^ (0.753290). MAPE varies from 0.1340% for B5→B6 to 1.8616% for C2→C3, and the highest R^2^ is obtained for B5→B6 (0.998516). These ranges show that the relative ranking of the tasks is metric-dependent and should be assessed using RMSE, MAE, MAPE, and R^2^ jointly.

Within the Tsinghua dataset, B4→B3 and B5→B6 have the same RMSE (0.002574), but B5→B6 yields a lower MAE (0.000063 versus 0.002076), a lower MAPE (0.1340% versus 0.2493%), and a higher R^2^ (0.998516 versus 0.996002). For the Oxford tasks, C1→C2 provides the lowest RMSE in the entire table, while C2→C3 shows a higher RMSE (0.003671), the highest MAPE (1.8616%), and a lower R^2^ (0.842800). Among the Tongji transfers, performance improves progressively from D1→D2 to D3→D4 and D5→D6: RMSE decreases from 0.016004 to 0.010683 and 0.003402, while R^2^ increases from 0.753290 to 0.989934 and 0.997023, respectively.

Overall, six of the eight tasks achieve R^2^ values of at least 0.9702 at their respective optimal calibration proportions, whereas D1→D2 and C2→C3 remain the more challenging transfers. The results therefore support selecting the target-domain calibration proportion separately for each transfer task instead of applying a universal ratio. They also demonstrate that strong cross-battery SOH estimation can often be achieved with limited target-domain data, although the required proportion and the resulting accuracy vary across source–target combinations.

## 5. Conclusions

To address the challenge of inaccurate battery state of health prediction under high-temperature conditions, this paper proposes a novel Ridge-Conv-Bi-LSTM framework with a dual-level transfer learning strategy. The model employs a Ridge regression baseline to capture the global degradation trend, which serves as a linear anchor for subsequent residual learning. A Conv-Bi-LSTM network is then trained to model the nonlinear residuals between the true SOH and this baseline, thereby enhancing the capacity to extract complex temporal dependencies. Furthermore, a dual-level transfer strategy—combining prior-regularized Ridge coefficient adaptation with lightweight fine-tuning of the pre-trained network—is introduced to enable rapid cross-battery adaptation with minimal target-domain calibration data. The main contributions of this study are summarized as HF extraction, experimental validation across single-battery, cross-battery and ablation scenarios, and robustness under high-temperature conditions. Four health-related features strongly correlated with capacity fade were extracted from raw cycling data. The locally weighted scatterplot smoothing algorithm was subsequently applied to denoise the features, effectively suppressing interference while preserving essential aging trends, thereby improving the reliability and quality of the training data. The proposed framework was evaluated using battery aging datasets from Tsinghua University, Tongji University and the University of Oxford. Experimental results demonstrate that the model consistently outperforms several established benchmarks—including Ridge, LSTM, Bi-LSTM, and RF—achieving substantial improvements in SOH estimation accuracy across both normal and high-temperature conditions.

Looking forward, several promising directions for future research can be identified. SOH estimation for battery packs and the modeling of cell-to-cell consistency could pave the way for a comprehensive, lifecycle-oriented battery health management system capable of adapting to diverse operational conditions, thereby offering more holistic technical support for advanced energy-storage applications.

## Figures and Tables

**Table 1:** Model hyperparameter settings for the single-battery extrapolation scenario.

Parameter	Value	Parameter	Value
Sliding window length	5	Training L2 regularization	8 × 10^−4^
Conv filters/size	32/3	Training max epochs	130
Bi-LSTM hidden units	64	Finetune initial learning rate	2 × 10^−4^
Fully connected units	32	Finetune L2 regularization	10^−3^
Dropout rate	0.12	Finetune max epochs	90
Training initial learning rate	7 × 10^−4^	Mini-batch size	16

**Table 2:** Model hyperparameter settings for the cross-battery scenario.

Parameter	Value	Parameter	Value
Sliding window length	6	Source L2 regularization	8 × 10^−4^
Conv filters/size	32/3	Source max epochs	120
Bi-LSTM hidden units	64	Target learning rate	2 × 10^−4^
Fully connected units	32	Target L2 regularization	10^−3^
Dropout rate	0.12	Target max epochs	80
Source learning rate	7 × 10^−4^	Mini-batch size	32

**Table 3:** SOH Estimation Performance at Optimal Proportions.

Dataset	Transfer	Percent (%)	RMSE	MAE	MAPE (%)	R^2^
Tsinghua	B2→B1	20	0.009349	0.008133	0.9568	0.9702
Tsinghua	B4→B3	20	0.002574	0.002076	0.2493	0.996002
Tsinghua	B5→B6	30	0.002574	0.000063	0.1340	0.998516
Oxford	C1→C2	10	0.001412	0.000000	0.4010	0.987381
Oxford	C2→C3	30	0.003671	0.000178	1.8616	0.842800
Tongji	D1→D2	5	0.016004	0.000019	1.0341	0.753290
Tongji	D3→D4	30	0.010683	0.000443	0.3294	0.989934
Tongji	D5→D6	30	0.003402	0.000017	0.2160	0.997023

## Data Availability

The Oxford Battery Degradation Dataset 1 is publicly available through the Oxford University Research Archive under DOI 10.5287/bodleian:KO2kdmYGg. The Tongji battery-aging dataset is publicly available through the Zenodo repository under DOI 10.5281/zenodo.6379165. The Tsinghua battery-aging data were generated by the authors’ team and have not been deposited in a public repository; these data are available from the corresponding author upon reasonable request. The processed health-related feature data and additional results generated in this study are also available from the corresponding author upon reasonable request.

## Supplementary Materials

The following supporting information can be downloaded at: https://www.mdpi.com/article/10.3390/ma19153332/s1. References [17,34] are cited in the Supplementary Materials.

## Abbreviations

Bi-LSTM	Bidirectional Long Short-Term Memory network
BMS	battery management systems
CNN	Convolutional Neural Network
Conv	Convolutional
DOD	depth of discharge
ECM	equivalent circuit models
EM	electrochemical models
HF	health-related feature
KAN	Kolmogorov–Arnold networks
LOESS	locally weighted scatterplot smoothing
LSTM	Long Short-Term Memory
MAE	mean absolute error
MAPE	mean absolute percentage error
NCA	Nickel Cobalt Aluminum
NCM	Nickel Cobalt Manganese
PSO	particle swarm optimization
R^2^	coefficient of determination
RF	random forest
RMSE	root mean square error
RNN	Recurrent Neural Network
SOH	State of Health
