# Supplementary material for: A Hybrid Ridge Regression–Convolutional Bidirectional Long Short-Term Memory Framework with Dual-Level Transfer Learning for State-of-Health Estimation of Lithium-Ion Batteries Under High Temperatures
Source: Materials (Basel). 2026 Aug 5;19(15):3332. doi: 10.3390/ma19153332 (PMC13467955; doi:10.3390/ma19153332)
Supplement: Supplementary file 1 [file materials-19-03332-s001.zip › materials-4439146-supplementary.pdf]

Supplementary Information

A hybrid Ridge regression-convolutional bidirectional long short-term memory framework with dual-level transfer learning for state-of-health estimation of lithium-ion batteries under high temperatures

Chengwei Ge [1,2], Chunling Wu [1,2,]*, Zhen Zhang [1,2,]*, Kaile Cao [1,2], Li Wang [3], Mingwei Gao [4], Xiangming He [3,]*

[1] School of Energy and Electrical Engineering, Chang'an University, Xi'an 710064, China; 2025138058@chd.edu.cn (C.G.); 2024901939@chd.edu.cn (K.C.)
[2] Shaanxi Key Laboratory of New Transportation Energy and Automotive Energy Saving, School of Energy and Electrical Engineering, Chang'an University, Xi'an 710018, China
[3] Institute of Nuclear and New Energy Technology, Tsinghua University, Beijing 100084, China; wang-l@tsinghua.edu.cn
[4] Xi'an Stropower Technologies Co., Ltd., Xi'an 710076, China; mingwei.gao@stropower.com
* Correspondence: wuchl@chd.edu.cn (C.W.); zhenzhang@chd.edu.cn (Z.Z.); hexm@tsinghua.edu.cn (X.H.)

1. Data Sources and Preprocessing

The experimental data utilized herein originate from three authoritative research institutions: the 6Ah lithium iron phosphate pouch cell cycling ageing experiments conducted by Tsinghua University's Institute of Nuclear and New Energy Technology, and the 740mAh lithium cobalt oxide battery cycling ageing experiments undertaken by the University of Oxford, as well as the 3500mAh lithium-ion battery cycling ageing experiments provided by Tongji University. The Tsinghua University dataset comprises 15 experimental groups categorized by varying test conditions, encompassing a total of 30 pouch-type lithium iron phosphate batteries. The Oxford University dataset covers eight lithium cobalt oxide battery samples. All experiments were conducted at a constant ambient temperature of 40°C, employing a data acquisition method involving comprehensive battery performance testing after every 100 cycles. The Tongji University dataset consists of 130 commercial 18650 lithium-ion batteries tested under three temperature levels—25°C, 35°C, and 45°C—providing a broader range of thermal conditions for model validation [1].

In battery cycling tests conducted at Tsinghua University, the rated capacity of the lithium iron phosphate pouch cell was 6Ah, with a rated voltage of 3.2V. The upper charging cut-off voltage was 3.65V, and the lower discharging cut-off voltage was 2.7V. In battery cycling experiments conducted at the University of Oxford, lithium cobalt oxide batteries had a rated capacity of 740mAh, a rated voltage of 3.7V, a charging upper limit cut-off voltage of 4.2V, and a discharging lower limit cut-off voltage of 2.7V. In the battery cycling experiments conducted at Tongji University, the 18650 lithium-

ion cells had a rated capacity of 3500mAh and a charging upper limit cut-off voltage of 4.2V; the discharging lower limit cut-off voltage was 2.65 V for NCA cells, 2.5 V for NCM cells, and 2.5 V for the combined NCA+NCM group.

The charge-discharge cycle test designed by Tsinghua University's Institute of Nuclear and New Energy Technology comprises five stages: constant-current charging, constant-voltage charging, post-charge rest period, constant-current discharging, and post-discharge rest period. The specific process is as follows: First, charge using constant-current mode until the voltage reaches the upper cutoff voltage of 3.65V; Subsequently, the mode switches to constant voltage charging, maintaining a constant voltage of 3.65V until the charging current decays to 30mA, at which point charging ceases. Next, the battery enters the resting phase: 30 minutes at room temperature or 2 hours at 50°C. Following this, constant current discharge is applied until the battery voltage reaches the lower cutoff voltage of 2.7V. Finally, the battery undergoes another resting period. The battery charge-discharge cycle aging test conducted by the University of Oxford includes constant-current charging and constant-current discharging phases, with the specific process as follows: First, charge at a 1C constant current to the upper cutoff voltage of 4.2V, then discharge at a 1C constant current to the lower cutoff voltage of 2.7V. The charge-discharge cycle test designed by Tongji University also comprises five stages: constant-current charging, constant-voltage charging, post-charge rest period, constant-current discharging, and post-discharge rest period.

To conduct an in-depth analysis of typical ageing characteristics, this study selected fourteen representative battery samples spanning three datasets. Samples B1 through B6 originate from the Tsinghua University dataset: B1 and B2 were cycled at 25°C with a 0.33C charging and 0.66C discharging strategy; B3 and B4 were tested at 50°C under a 0.1C charging and 0.33C discharging protocol at 100% depth of discharge (DOD); B5 and B6 were also tested at 50°C with the same current rates but at 80% DOD. Samples C1 to C3 belong to the University of Oxford dataset and were cycled at 40°C with 100% DOD. Samples D1 to D6 are drawn from the Tongji University dataset and were all tested under a 0.5C charging and 1C discharging strategy at 100% DOD, with D1 and D2 assigned to 25°C, D3 and D4 to 35°C, and D5 and D6 to 45°C.The detailed test parameters for each sample are summarized in Table.S1. The selected dataset systematically covers key characteristic parameters during the battery ageing process, including voltage, current, capacity, charging duration, and discharging duration.

2. Supplementary table

Table S1. Experimental battery parameters

battery	DOD（%）	charge current（A）	discharge current（A）	temperature（℃）
B1	100	2	4	25

B2	100	2	4	25
B3	100	0.6	2	50
B4	100	0.6	2	50
B5	80	0.6	2	50
B6	80	0.6	2	50
C1	100	0.74	0.74	40
C2	100	0.74	0.74	40
C3	100	0.74	0.74	40
D1	100	1.75	3.5	25
D2	100	1.75	3.5	25
D3	100	1.75	3.5	35
D4	100	1.75	3.5	35
D5	100	1.75	3.5	45
D6	100	1.75	3.5	45

3. Supplementary figures

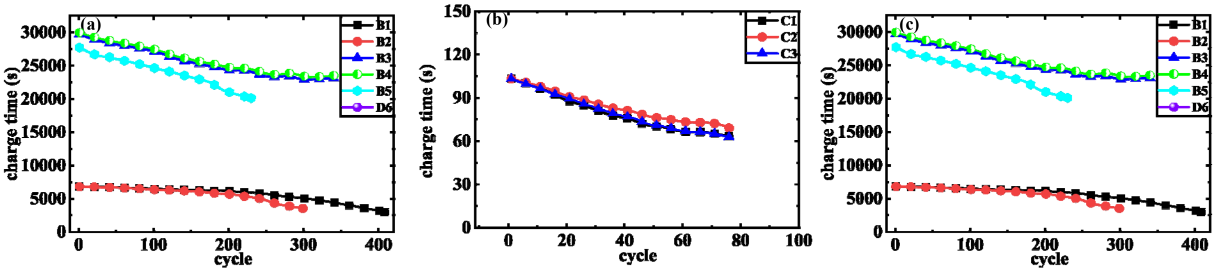

Figure S1. Equal voltage rise charge time-cycle count: (a)B1-B6; (b)C1-C3; (c)D1-D6.

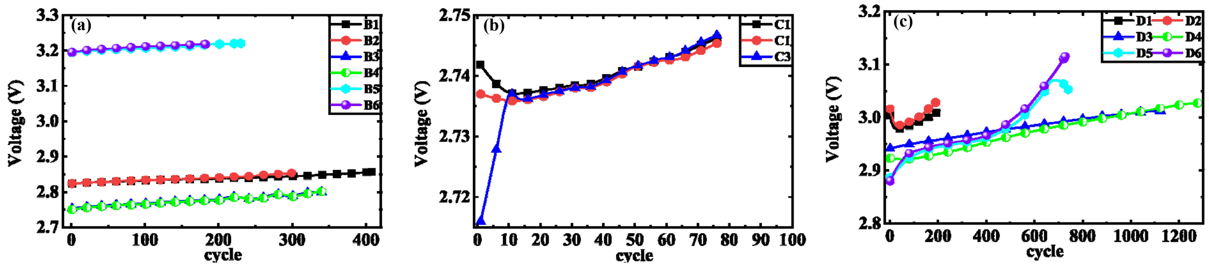

Figure S2. Constant-current discharge termination voltage-cycle count: (a)B1-B6; (b)C1-C2; (c)D1-D6.

4. The workflow of LOESS

1）For the target point "x_0", select the neighborhood radius to determine the set of neighborhood points:

$$N(x_0)=\{x_i \,||x_i - x_0| \le d\} \tag{S1}$$

2）Calculate the weights for each point (using a cubic kernel function):

$$\omega_i(x_0)=\left(1-\left|\frac{x_i - x_0}{d}\right|^3\right)^3 \tag{S2}$$

3）Local polynomial fitting:

$$\hat{f}(x)=\beta_0+\beta_1 x+\beta_2 x^2+\cdots+\beta_p x^p \tag{S3}$$

4) Weighted least squares coefficient solution:

$$\min_{\beta} \sum_{i \in N(x_0)} \omega_i(x_0)\left[y_i - \hat{f}(x_i) \right] \tag{S4}$$

5) Target point estimation:

$$\hat{y}_0 = \hat{f}(x) = \beta_0 + \beta_1 x_0 + \beta_2 x_0^2 + \cdots + \beta_p x_0^p \tag{S5}$$

5. Correlation Analysis of the Selected Health-Related Features

Table S2. Pearson correlation coefficients between the selected health-related features and battery capacity for the cells used in this study.

battery	HF1	HF2	HF3	HF4
B1	0.9939	-0.9961	-0.9881	0.9923
B2	0.9880	-0.9986	-0.9918	0.9928
B3	0.9948	-0.9636	-0.9802	0.9896
B4	0.9949	-0.9657	-0.9820	0.9909
B5	0.9979	-0.9968	-0.9972	0.9979
B6	0.9963	-0.9925	-0.9938	0.9963
C1	0.9977	-0.7976	-0.7993	0.9963
C2	0.9973	-0.9468	-0.9441	0.9958
C3	0.9978	-0.8749	-0.8748	0.9956
D1	0.9973	-0.9630	-0.9522	0.9968
D2	0.9988	-0.9821	-0.9253	0.9985
D3	0.9851	-0.9974	-0.9935	0.9764
D4	0.9831	-0.9961	-0.9878	0.9818
D5	0.9699	-0.9645	-0.7699	0.9742
D6	0.9761	-0.9701	-0.7997	0.9779

6.LSTM computation process

The LSTM comprises four constituent components: the input gate, the forget gate, the output gate, and the memory unit. Its computational process is described by equation (S6) [2]:

$$\begin{cases} f_t = \sigma(W_f \cdot [h_{t-1}, x_t] + b_f) \\ i_t = \sigma(W_i \cdot [h_{t-1}, x_t] + b_i) \\ o_t = \sigma(W_o \cdot [h_{t-1}, x_t] + b_o) \\ \tilde{c}_t = tanh(W_c \cdot [h_{t-1}, x_t] + b_c) \\ c_t = f_t * c_{t-1} + i_t * \tilde{c}_t \\ h_t = o_t * tanh(c_t) \end{cases} \tag{S6}$$

In the formula, W_f is weight of the forgetful gate; W_i is weight of input gate; W_c is weight of candidate memory unit; W_o is weight of output gate; $[h_{t-1},x_t]$ is the connection between the hidden state h_{t-1} from the previous time step and the input x_t at the current time step; b_f、b_i、b_c、b_o are the bias term with the activation function; tanh is the hyperbolic tangent activation function; * is the Hadamard operator [2].

References

[1] Zhu J, Wang Y, Huang Y et al. Data-driven capacity estimation of commercial lithium-ion batteries from voltage relaxation. Nat Commun 13, 2261 (2022).

[2] Si M T. Research on lithium-ion battery health feature extraction and SOH prediction[D]. Anhui University of Science and Technology, 2025.
